# Comparative efficacy of non-vascularized and vascularized bone grafts, with emerging insights into bone biomaterial grafts, in scaphoid fracture nonunion treatment: A systematic review and meta-analysis

**DOI:** 10.1016/j.jot.2025.06.009

**Published:** 2025-06-28

**Authors:** Reza Karimnazhand, Roshanak Shams, Ali Behmanesh, Masoud Vosough, Azadeh Gharooee Ahangar, Laura Serrano Barrenechea, Omid Mahmoudinasab, Farid Najd Mazhar

**Affiliations:** a Bone and Joint Reconstruction Research Center, Department of Orthopedics, School of Medicine, Iran University of Medical Sciences, Tehran, Iran; b Department of Regenerative Medicine, Cell Science Research Center, Royan Institute for Stem Cell Biology and Technology, ACECR, Tehran, Iran; c Experimental Cancer Medicine, Institution for Laboratory Medicine, Karolinska Institute, Stockholm, Sweden; d Physical Medicine and Rehabilitation Specialist, Rehabilitation Medicine Department, Southern Älvsborg Hospital, Borås, Sweden

**Keywords:** Scaphoid fractures, Nonunion, Vascularized bone graft, Non-vascularized bone graft, Bone biomaterials, Meta-analysis, Orthopedic surgery

## Abstract

**Background:**

Scaphoid fractures are prevalent wrist injuries with significant treatment challenges, especially when get complicated by nonunion and avascular necrosis. Various grafting techniques, including non-vascularized bone grafts (NVBGs), vascularized bone grafts (VBGs), and bone biomaterial grafts, are utilized to promote healing, but the comparative efficacy of these methods remains unclear.

**Objective:**

This systematic review and meta-analysis aim to assess the efficacy of different types of bone grafts (NVBGs, VBGs, and bone biomaterials) in the treatment of scaphoid nonunion, focusing on outcomes including union rates, time to healing, and functional recovery scores.

**Methods:**

A systematic search of PubMed, Scopus, Cochrane and Web of Science was conducted to identify eligible studies published between 2000 and 2024. studies were categorized into: (1) comparative studies of NVBG vs. VBG, (2) studies reporting only NVBG outcomes, and (3) studies reporting only VBG outcomes. Bone Biomaterials were assessed separately due to limited data (one study). Meta-analyses were performed on in common reported outcomes for each group including union rates, time to healing, range of motion, grip strength, and Modified Mayo Wrist Scores (MMWS).

**Results:**

A total of 62 studies involving 2332 scaphoid nonunion patients were included. VBGs demonstrated significantly higher union rates and shorter healing times compared to NVBGs. VBG-treated patients also showed better functional outcomes, including greater grip strength and MMWS in comparative studies NVBGs were less effective in evaluated cases and required longer healing times. The use of bone biomaterial grafts, although limited in the current literature, showed promising results comparable to NVBGs, but further studies are needed.

**Conclusion:**

VBGs demonstrated higher union rates and shorter healing times compared to NVBGs, with better functional outcomes in some cases, though evidence certainty is moderate. Bone biomaterials represent a promising alternative to traditional grafts but require more evidence to support their widespread use. Treatment decision-makers should consider clinical context and case-specific conditions. Further research is needed to confirm these trends.

Translational potential: This study helps clinical decision-making by evaluating the efficacies of different bone grafts outcomes in complex scaphoid nonunions, potentially reducing treatment failures. It also underscores the emerging role of bone biomaterial grafts as a less invasive alternative, paving the way for personalized orthopedic strategies needing further evaluations to be used for treating scaphoid nonunions.

## Graphical abstract

Different types of gone grafts resources applicable for scaphoid nonunion fractures treatment. Figure depicts the overall idea of this research regarding the applicability and most commonly used sources of each bone grafts type.

**Figure:**
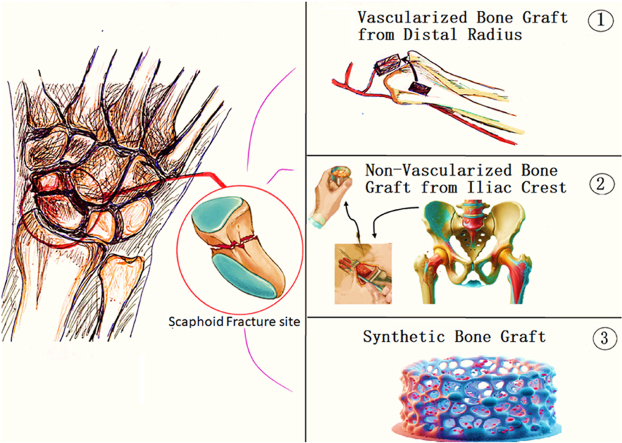

## 1 Introduction

Scaphoid fractures account for a significant portion of wrist injuries and present substantial treatment challenges, particularly due to their susceptibility to nonunion and avascular necrosis [1]. The risk of nonunion in scaphoid fractures, a prevalent complication, significantly complicates treatment outcomes and patient recovery. This nonunion risk is heightened by the scaphoid's unique anatomical and vascular characteristics; its blood supply is primarily retrograde, entering Distally and rendering the proximal segment particularly vulnerable to ischemia following fracture [2]. Factors correlated to the increased risk of nonunion include late diagnosis, inappropriate immobilization, and the fracture's site within the scaphoid. Most of the times, fractures of proximal pole have high risk to lead nonunion due to their poor vascular supply. Treatment of these types fractures needs a deep understanding of both the biological and biomechanical basics of bone healing and defining the role of surgical intervention in reaching optimal outcomes [3]. The effectiveness of various grafting techniques—non-vascularized, vascularized, and Bone biomaterial grafts—has been a focal point of research in orthopedic practice. Surgical management of scaphoid nonunion with bone grafting, while effective, carries varying complication rates depending on the technique employed. Non-vascularized bone grafts (NVBGs), commonly sourced from the iliac crest or Distal Radius, are associated with donor site morbidity in 5–10 % of cases, including persistent pain, hematoma, or infection, alongside a 2–5 % risk of graft failure due to inadequate vascularization [4,5]. Vascularized bone grafts (VBGs), such as those from the Distal Radius or medial femoral condyle, offer improved healing potential but increase surgical complexity, resulting in higher complication rates, including donor site issues (e.g., numbness, wound dehiscence) and postoperative infection or vascular pedicle compromise [3]. In Addition, VBGs are of higher technical challenges and require more operation facilities. Hence, surgeons should be cautious in choosing VBGs for scaphoid non-union treatment [6]. There is a lack of definitive evidence to guide treatment choices concerning the relative efficacy of these techniques. Non-vascularized bone grafts (NVBG) are the traditional choice for simpler cases of scaphoid nonunion, where the existing blood supply is deemed adequate [7]. The mentioned bone substitutes, which are mostly harvested from the iliac crest or Distal Radius of the patient, is dependent on the surrounding vascular supply to heal and promote bone regeneration [5]. Other sources of NVBG can be rib or tibial grafts, which are less commonly used but provide additional alternatives depending on the condition of the patient and surgeon's preference [2]. On the other hand, Vascularized bone grafts (VBG) are increasingly gotten attention in more complex cases with impaired blood supply to the scaphoid [8]. These grafts are transplanted with their own blood vessels, which theoretically lead to more robust and reliable bone tissue healing. Typical resources for VBGs include the 1,2 intercompartmental supraretinacular artery graft from the Radius providing their own specific characteristics in terms of size, shape, and vascular supply [9]. Other less prevalent but notable sources include grafts from the lateral femoral condyle or the second metatarsal [10,11], which may be chosen based on specific anatomical and surgical considerations. However, using autologous bone grafts face critical challenges including difficulties in availability of the bone resources, donor site morbidity, and prolonged surgical time [4]. These challenges have pushed advancements in biomedical research, specifically in the development of synthetic or natural bone biomaterial graft substitutes for reconstructing different bone defects. Application of bone biomaterials for the treatment of bone fractures, particularly nonunions represents a promising modality in hand orthopedic surgery [12]. Synthetic or natural bone biomaterials offer several advantages over the traditional autografts and allografts, including the elimination of donor site morbidity, consistent quality, and the potential for customization to meet specific clinical needs. Today, bone tissue engineering science is emerging to replace the gold standard of autografts by delivering a biocompatible bone scaffold that mimics natural bone, inducing osteogenesis within an osteoconductive environment. These can be achieved by incorporating growth factors and stem cells into specific materials to enhance cellular development and bone regeneration and promoting proper vascularization to meet the functional nutritional of the formed bone tissues [13]. Although these modern grafts are a novel and evolving treatment modality with a limited evidence base, primarily due to their recent introduction and the time required for translational research to yield clinical studies, but their ability to support bone regeneration and integration without the need for donor tissue harvesting can be valuable regarding the morbidity risk of the donor sites [14]. Various biocompatible materials such as calcium phosphate ceramics, bioactive glass, and polymer-based composites can be used as bone substitutes. Each biomaterial has unique properties that can mimic the biomechanical and biological characteristics of bone, potentially reducing the risk of infection and donor site morbidity. Recent advancements have also proved the incorporation of growth factors and other bioactive agents to enhance osteogenesis and angiogenesis directly at the fracture site [13]. Application of bone biomaterials for the treatment of bone fractures, particularly nonunions represents a promising modality in hand orthopedic surgery [12]. Synthetic or natural bone biomaterials offer several advantages over the traditional autografts and allografts, including the elimination of donor site morbidity, consistent quality, and the potential for customization to meet specific clinical needs. Several clinical trials has been performed to evaluate the effectiveness of different kinds of biomaterials in treatment of small bone defects formed post osteomyelitis and benign tumors resection [15]. However, these grafts may elicit inflammatory responses due to foreign body reactions or suboptimal biocompatibility. Additionally, variable resorption rates and their availability pose another concerns, as some biomaterials degrade too rapidly to provide sustained structural support, while others persist excessively, hindering natural bone remodeling [14,15]. Availability of these grafts is further constrained by stringent regulatory approval processes, which demand extensive preclinical and clinical data to ensure safety and efficacy, often prolonging time-to-market [12]. Despite of these limitations, in the specific context of scaphoid fractures specially in cases of nonunion, bone biomaterial grafts can be particularly beneficial. Designing these grafts in order to release specific growth factors such as BMP-2 (Bone Morphogenetic Protein 2) or VEGF (Vascular Endothelial Growth Factor) to stimulate bone growth and enhance vascularization at the fracture site is of great interest [13].

By comparing the outcomes of different bone grafting sources through a comprehensive systematic review and meta-analysis, this study aims to propose clear, evidence-based guidelines for the treatment of scaphoid fractures. The review not only assessed the direct clinical outcomes such as union rates and time to healing among different types of bone grafts, but also consider patient-specific outcomes including functional performances, and overall satisfaction, thereby guiding comprehensive treatment recommendations for various clinical scenarios.

## 2 Methods

This systematic review was conducted following the Preferred Reporting Items for Systematic Reviews and Meta-Analyses (PRISMA) guidelines [16]. A comprehensive systematic literature search was performed in PubMed, Scopus, Web of Science, and the Cochrane Library from 2000 to April 2024, using tailored strategies outlined in Table 1. No other supplementary papers were identified through non-systematic searches of pertinent websites. Two independent investigators performed a three-stage systematic screening of retrieved articles. Studies included in this analysis adhered to the criteria outlined by the Population, Intervention, Comparison, and Outcome (PICO) framework for the research question: "Do surgical outcomes differ in terms of healing rate and functional outcomes when non-vascularized or vascularized bone grafts or biomaterials were used for non-union fractures of the scaphoid bone?" In the first stages, the two researchers independently checked the titles and keywords of retrieved articles to rate their eligibility based on inclusion criteria. Next, abstracts were reviewed for relevance to the study topic using predefined inclusion criteria. In the third stage, the same researchers read and fully analyzed the full text of screened articles according to eligibility criteria. In cases of disagreement, agreement was reached through a third reviewer. If full texts were not available, authors were contacted for access. Qualitative and quantitative data regarding the outcomes of different graft types and scaffold materials on the healing of non-union scaphoid fractures were extracted and categurized. Data retrived from final texts included publication year, country of origin, graft and scaffold types, union rates, time to union, range of motion (ROM), grip strength, Visual Analog Scale (VAS) scores, and Modified Mayo Wrist Scores (MMWS).

**Table 1:** The search strategy through 4 databases.

Database	Search strategy
PubMed	("Scaphoid Nonunions" [TIAB] OR "Scaphoid bone" [TIAB] OR "scaphoid fracture" [TIAB]) AND (graft∗[TIAB] OR biomaterial[TIAB] OR scaffolds[TIAB] OR "Mesenchymal Stromal Cells" [TIAB])
Scopus	TITLE-ABS-KEY ("Scaphoid Nonunions" OR "Scaphoid bone" OR "scaphoid fracture") AND TITLE-ABS-KEY (graft∗ OR biomaterial OR scaffolds OR "Mesenchymal Stromal Cells")
Web of sciences	TS= (("Scaphoid Nonunions" OR "Scaphoid bone" OR "scaphoid fracture") AND (graft∗ OR biomaterial OR scaffolds OR "Mesenchymal Stromal Cells"))
Cochrane Library	("Scaphoid Nonunions" OR "Scaphoid bone" OR "scaphoid fracture") AND (graft∗ OR biomaterial OR scaffolds OR "Mesenchymal Stromal Cells")

### 2.1 Inclusion and exclusion criteria

This systematic review included studies evaluating the efficacy of non-vascularized bone grafts (NVBGs), vascularized bone grafts (VBGs), or bone biomaterial grafts for treating scaphoid nonunion, encompassing randomized controlled trials, cohort studies, case series, and case–control studies that reported outcomes such as union rates, time to union, functional outcomes (e.g., ROM, grip strength and MMWS. Studies were required to be published in English between 2000 and 2024 and involve human subjects, with full-text availability either directly or upon author request. studies using usual screw fixation tools as a stabilizing technique (for example Herbert screws) for bone grafts were included, provided their primary focus was on VBG or NVBG outcomes (and not concentrated on the fixation screws or methods specifically the studies with possible industrial intentions).

Excluded were animal studies, review articles, case reports, editorials, and studies not reporting relevant outcomes or those focusing on fixation methods rather than the bone graft type (such as specific screws or fixation devices), other types of bone grafts or additional treatments beyond those specified as well as those lacking accessible full texts after attempts to contact authors failed.

### 2.2 Quality evaluation and risk of bias assessment

The quality of studies methods was independently checked by two investigators (RS and RK), in accordance with Baker et al. [17]. Using the Cochrane ROB2 for randomized control trials and ROBINS-I for non-randomised studies - of Interventions [18], the risk of bias was assessed based on defined domains including the randomization process, blinding (deviations from the intended), missing data, outcome measurement and selective or multiple outcome reporting. The results were visualized using ROBVIS tool [19]. The certainty of evidence for key outcomes (union rates, time to union, functional scores) was assessed using the GRADE approach, following PRISMA 2020 recommendations. We evaluated risk of bias, inconsistency, indirectness, imprecision, and publication bias, starting with High certainty for RCTs and Low for observational studies, adjusting as necessary.

### 2.3 Data analysis

The outcomes which were appropriately reported in different studies, including bone healing rate, time to union, ROM, Grip strength, MMWS scores were categorized and analyzed. For comparative studies, meta analyses performed comparing Mean differences for each outcome between VBG and NVBGs. For other cohort or case series which only evaluated the outcomes of one on the considered grafts, a pooled integrated value was calculated using random effect size calculation method. Statistical analyses were conducted to synthesize outcomes across studies, employing random-effects models to account for anticipated clinical and methodological heterogeneity among trials, following the DerSimonian-Laird method implemented in Review Manager (RevMan) Version 5.3. Heterogeneity was assessed using the I^2^ statistic, where values of 0–25 % indicated low heterogeneity, 25–70 % moderate, and >70 % high, complemented by the Tau^2^ statistic to quantify between-study variance; forest plots visualized these results for each outcome (e.g., union rates, time to union, ROM and MMWS). For comparative studies directly evaluating NVBGs versus VBGs, effect sizes were calculated as mean differences (MDs) with 95 % confidence intervals (CIs) for continuous outcomes (e.g., time to union, MMWS) and risk ratios (RRs) with 95 % CIs for dichotomous outcomes (e.g., union rates), facilitating direct treatment comparisons. In contrast, pooled estimates from non-comparative studies (e.g., NVBG-only or VBG-only cohorts) were derived using weighted means with standard deviations, integrating data via random-effects modeling to provide overall outcome summaries.

### 2.4 Standardized reporting protocol

To ensure consistency, reproducibility, and comparability across studies on scaphoid nonunion treatments, the following standardized reporting protocol outlying core outcome measures, assessment methods, and recommended follow-up intervals was used:

Union Rates is defined by the radiographic evidence of bone bridging across the fracture site in two or more views. Independent radiographic assessment done by at least two blinded reviewers. The results reported with Percentage of total cases and the exact number of cases. Time to Union**,** the duration from the surgical intervention to radiographic evidence of bone union, calculated in weeks, with evaluations at standard follow-up intervals (e.g., 6, 12, 24 weeks) and reported as Mean and standard deviation. Grip strength assessed using a Jamar dynamometer or equivalent, standardizing as a percentage of the contralateral (uninjured) hand were considered. **ROM,** reported as total wrist flexion-extension range using a goniometer. **MMWS** were reported as total score of Mean values with standard deviations for each functional outcome and the measurement tools used. Patient-Reported Outcomes including VAS for pain and DASH questionnaire as Mean scores with standard deviations were considered.

## 3 Results

**Fig. 1:**
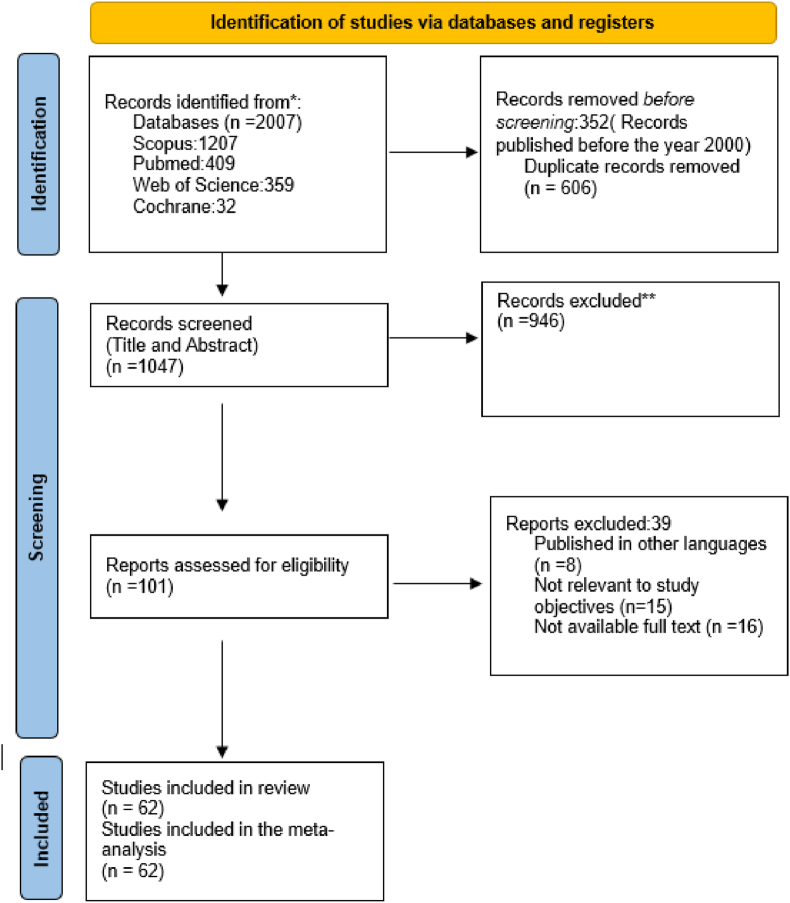
The PRISMA diagram of the included and excluded studies.

Based on the defined search strategy, a total of 2007 references including 359 articles from WoS, 1207 papers from Scopus, 32 studies from Cochrane and 409 articles from PubMed were retrieved (up to April 2024). 352 studies were removed before screening due to being published before 2000. Fig. 1 depicts the PRISMA 2020 flow diagram of the studies selection stages. After precise evaluations, a total of 62 final studies were included in the study. We divided the final articles based on their study types into 3 groups [1]: comparative studies of NVBG vs. VBG [2], studies reporting only NVBG outcomes, and [3] studies reporting Bone biomaterials outcomes. Bone Biomaterials were assessed separately due to limited data (one study). Totally, data from 2332 patients were included. The mean age of the participants was 30.12 years. Among those with available gender information, 88.01 % were male and 11.99 % were female. Regarding the location of fractures, 503 patients (27.82 %) had fractures at the proximal pole (PP), 1210 patients (66.88 %) had fractures at the waist (W), and 96 patients (5.30 %) had fractures at the distal (D) site. The average nonunion time was 27.33 months, and the mean time to healing for all patients was 18.29 weeks. 15 studies compared different bone grafts outcomes including 10 studies comparing VBG vs NVBG. Other case series or cohort studies were also categorized based on applying only VBG (18 articles) or NVBG (34 articles). Studies with reported quantitative data were used for statistical analysis. Weighted means and standard deviations were calculated to summarize the values reported in the individual studies and to compare them.

### 3.1 Comparative studies (VBG vs NVBG)

Among the 10 studies comparing the outcomes of VBGs (mostly from Distal Radius) with NVBGs (mostly from iliac crest and Distal Radius) (Table 2), data from 577 scaphoid non-union patients who underwent surgery were included in the analyses. Of these, 286 patients received iliac crest or Distal Radius bone grafts as NVBGs (58 DR and 228 IC), while 246 patients were treated with VBGs mostly from the Distal Radius (14 with other sources). A limited number of studies used different sources of bone grafts, such as the medial femoral condyle for VBGs and the Distal Radius for NVBGs which were not enough to be meta-analyses as subgroups due to low sample size and were included in the total evaluations. Table 2 demonstrates all the comparative studies and their extracted data. A total of 405 patients with gender information were included in the study, comprising 353 men (87.16 %) and 52 women (12.84 %). The average age of the patients was 27.18 years. The average nonunion time was 26.39 months, and the average time to healing for all patients was 18.44 weeks. Regarding the cite of fractures, 29 (9.35 %) were proximal pole (PP), 255 (82.25 %) were waist (W), and 26 (8.40 %) were distal (D).

**Table 2:** Characteristics of Comparative studies.

Author	Year	Country	Sample size	Mean age	Sex M/F	AVN	Nonunion time	Cite of scaphoid fracture	Graft type	Graft source	Operation time	Number of healed	Time to Healing	ROM (F/E)	Grip Strength	MMWS	DASH	VAS
**Braga silva** [20]	2008	Brazil	Vascularized radial grafts:35	26.7	24M\11F	No	30	23w/12d	VBG	Distal Radius	NA	32	8	56.5°/47.7°	60 %	NA	NA	NA
			Non-vascularized iliac grafts:45	25.2	32M/13F		33.6	33w/12d	NVBG	Iliac Crest		45	8.9	49.8°/47.4	59 %			
**Ribak** [21]	2010	Brazil	Vascularized bone graft:46	NA	NA	Yes	25.3	21pp/25w	VBG	Distal Radius	NA	41	9.7	NA	NA	NA	NA	NA
			Non-vascularized bone graft:40				22.5	16pp/22w/2d	NVBG	Distal Radius		29	12					
**Bao** [22]	2013	China	Pedicled vascularized bone graft:7	28	12M/1F	No	12.5	pp	VBG	Lister's tubercle	NA	7	NA	124°	93 %	86.4	NA	NA
			Traditional bone graft:6						NVBG	Iliac Crest		5		94°	75 %	717		
**Caporrino** [23]	2014	Brazil	VBG:35	26.1	33M/2F	No	36.9	2pp/23w	VBG	Distal Radius	NA	31	8	79.1°	89.2 %	NA	NA	NA
			NVBG:38	29.1	38M/2F				NVBG			32	10	76.3°	86.1 %			
**Hirche** [24]	2017	Germany	VBG:28	29.8	25M/3F	Yes	54	—	VBG	Distal Radius	112	21	NA	106°	85.3 %	71.1	NA	NA
			NVBG:45	27	43M\2F	No	22.9	—	NVBG	Iliac Crest	78	37		115°	89.3 %	77		
**Aibinder** [25]	2017	USA	ICBG:31	29.4	26M/2F	No	15.6m	30w/1p	NVBG	Iliac Crest Bone Graft	NA	22	18.7	51.0°/45.8°	36.1	NA	NA	NA
			MFC:45	22.8	7M	Yes		38w/7p	VBG	Medial femoral condyle		40	15.5	50.8°/42.9°	38.9			
			1,2-ICSRA:33	23.1	9M	Yes		12w/21p	VBG	1,2-Intercompartmental Supraretinacular Artery		28	25.6	48.2°/44.0°	40.2			
**Marasli** [26]	2021	Turkey	IC-NBG:8	28.9	6M/2F	No	NA	w	NVBG	Iliac Crest	NA	7	NA	73.1°/61.6°	90.6 %	83.7	NA	10
			MFC:7	28.4	7M				VBG	Medial femoral condyle		6		55.1°/42.2°	101 %	72.8		17.1
			1,2-ICSRA-VBG:9	33.9	9M				VBG	Distal Radius		9		65.4°/61.8°	110.5 %	72.7		30
**Fan** [27]	2023	Canada	VBG group:20	22	17M/3F	No	17	7pp/waist	VBG	Distal Radius	NA	19	16	57°/51°	96 %	NA	5	NA
			NVBG group:18	23	18M		18	2pp/waist	NVBG	Iliac Crest		21	17	52°/50°	84 %		6	
**Özdemir** [28]	2023	Turkey	Pronator quadratus vascularized pedicled muscle flap group: 16	24.4	NA	Yes	24.8	6pp/10w	VBG	Distal Radius	NA	15	12.0	62.67°/39.33°	35.7	80.9	NA	18.7
			autologous bone graft group: 24	28.3			30.4	9pp/15w	NVBG	Iliac Crest		19	12.7	63.54°/48.33°	42	79.5		21.7
**Shin** [9]	2024	South Korea	VBG group: 15	28	M	No	NA	NA	VBG	Distal Radius	114	15	NA	0	86.4 %	95	5.2	NA
			NVBG group: 23	26					NVBG	Iliac Crest	98	22		2	85.4 %	90	5.5	

In this table, 1,2-ICSRA stands for the "1st and 2nd Intercompartmental Supraretinacular Artery," IC-NBG refers to the "Iliac Crest Non-Vascularized Bone Graft," and MFC stands for the "Medial Femoral Condyle." Additionally, in the study by Shin, "Range of Motion Restriction" is used instead of "Range of Motion."

### 3.2 Studies evaluating NVBGs outcomes

Data from 34 studies which used the NVBGs from iliac crest or Distal Radius were retrieved and analyzed (Table 3). The total number of 1328 patients were treated with NVBG (Iliac crest 1,125, 203 other sources) and average age was 30.13 years. Among the patients for whom gender information was available, there were 1085 men (88.64 %) and 139 women (11.36 %). The average time to nonunion was calculated to be 23.29 months. Regarding the types of fractures, the distribution is as follows: the number of proximal pole fractures (pp) was 261 (22.77 %), the number of waist fractures (w) was 822 (71.73 %), and the number of Distal fractures (d) was 63 (5.50 %). Table 3 includes the characteristics and retrieved data from all studies which used NVBG for scaphoid non-union treatment.

**Table 3:** Characteristics of NVBG studies.

Author	Year	Country	Sample size	Mean age	Sex M/F	AVN	Nonunion time	Cite of scaphoid fracture	Graft source	Operation time	Number of healed	Time to Healing	ROM (F/E)	Grip Strength	MMWS	DASH	VAS
**Bullens** [29]	2005	Netherland	33	34	33M	NO	13	6 pp/7w	Iliac Crest	NA	29	13	92 %	97 %	NA	NA	12
**Tambe** [30]	2006	UK	Group A:44	31	NA	NA	NA	9 pp/30w/5 d	Iliac Crest	NA	NA	NA	NA	NA	NA	NA	NA
			Group B:24					4 pp/18 w/2d									
**Yasuda** [31]	2007	Japan	28	28	24M/4 F	NO	22	3 pp/19w/6 d	Distal Radius	NA	28	7	77°/79°	93 %	NA	NA	NA
**Schreuder** [32]	2008	Belgium	16	32.3	NA	NO	27	NA	Iliac Crest	NA	13	NA	76.4 %/79.1 %	84.9 %	NA	17	NA
**Megerle** [33]	2008	Germany	31	29	31M	NO	15	NA	5 Iliac crest/26 Distal Radius	NA	21	16	80°/73 %	34Kg/80 %	73	17.8	NA
**Huange** [34]	2009	China	49	30.6	43M/6 F	NO	19.8	3pp/41w /5d	Iliac Crest	NA	46	50.4	75°/79°	96.3 %	NA	NA	NA
**Reigstad** [35]	2010	Norway	81	29	69M/12 F	NO	36	25 pp/53w/3d	Iliac Crest	NA	72	NA	NA	NA	NA	NA	NA
**Ghoneim** [36]	2011	Egypt	14	26	14M	YES	16.5	14w	Iliac Crest	NA	13	15.2	102°/78.7 %	NA	73	NA	NA
**Matsuki** [37]	2011	Japan	11	20.3	11M	2 AVN	12.4	11pp	Iliac Crest	NA	11	17.7	NA	NA	91.4	NA	NA
**Cohen** [38]	2013	USA	12	22	12M	YES	11	12w	Lister tubercle	NA	12		71°/61° 81 %/68 %	103 %	88	4	3
**Garg** [39]	2013	India	Distal Radius:42	32.4	25M/17F	NO	54	10 pp/32w	Distal Radius	NA	37	16.8	75.5°/82.2°	44 Kg	NA	NA	NA
			Iliac crest:46	36.8	30M/16F		64.8	12 pp/34w	Iliac Crest		40	18	75°/81.5°	40 Kg			
**Park** [40]	2013	Korea	Group A:34	34	61M\ 4F	5 AVN	28.7	7pp/27w/5d	Iliac Crest	NA	30	14.5	58.2°/58.5°	NA	77.1	NA	NA
			Group B:31			5 AVN		11pp/19w/1d			26		59.5°/55°		80		
**Kamrani** [41]	2014	Iran	11	31	11M	NO	14	11pp	9 Iliac crest/2 Distal Radius	NA	10	14.4	139°	39 Kg	76	NA	NA
**Ciraki** [42]	2016	Turkey	89	30.2	81M/7F	2 AVN	17.8	27pp/60w/2d	54 Iliac wing/35 Distal Radius	NA	71	16.9	NA	35.7 Kg	78.1	NA	NA
**Han** [43]	2017	Republic of Korea	30	32.8	28M/2F	NO	10	30w	Iliac Crest	NA	30	12.5	76°/73°	NA	93.5	NA	NA
**Conget** [44]	2017	France	23	26	20M/3F	NO	17.3	1pp/22w	Distal Radius	56	23	16	119°	41 Kg	NA	NA	15
**Dustmann** [45]	2017	NA	52	30.9	48M/4F	NO	35	14pp/37w/1d	Iliac Crest	NA	35	NA	115.3°	93 %	91.2	9.2	NA
**Kiran** [46]	2017	Scotland	GroupA: 29	27.6	48M/2 F	NO	15.9	3pp/17w/1d	40 Iliac crest\10 Dital Radius	NA	22	NA	NA	NA	NA	NA	NA
			GroupB: 21					4pp/23w/2d			16						
**Kim** [47]	2018	Republic of Korea	24	32.5	21M/3 F	YES	32	10pp/14w	Iliac Crest	NA	22	NA	86°	38 Kg	70	NA	NA
**Mani** [48]	2018	Nepal	55	28.5	31M/4 F	NO	7.7	6pp/24 w/15d	31 Distal Radius/14 Iliac crest	75	42	13.1	132.8°	31.1 Kg	84.4	NA	NA
**Schormans** [49]	2020	Netherland	49	31	46M/3F	6 AVN	43.2	—	Iliac crest	NA	47	18	124°	79 %	NA	NA	NA
**Yeh** [50]	2020	Taiwnan	18	32.7	13M/5 F	NO	20.8	w	Iliac Crest	NA	18	14.3	86 %	86 %	84.4	12.4	NA
**Hegazy** [51]	2021	Kingdom of Saudi Arabia	C-Only Group: 49	33.4	36M/13F	NO	27.9	w	Iliac Crest	NA	44	24	93 %	95 %	NA	NA	7
			CC Group: 49	33.2	39M/10F		28.3				46		95 %	95 %			6
**Hsiung** [52]	2021	Taiwan	41	29.9	40M /2 F	NO	11.2	9 pp/32 w/1d	Distal Radius	116	38	18.4	81.5°/64.5°	74.7 %	94	NA	2
**Arik** [53]	2022	Turkey	16	25.1	M	NO	32	w	Distal Radius	NA	14	18	49°/54°	82.5 %	82.3	NA	NA
**Estrella** [54]	2022	Philippine	12	27.4	M	NO	9.5	3pp/9	Iliac Crest	NA	11	11.5	82°/81°	82 %	NA	NA	13
**Lee** [55]	2022	Republic of Korea	15	44	14M/1F	NO	12	3pp/12 w	Iliac Crest	155	15	9.7	47°/59.2 %	88.6 %	80	NA	13
**Waitayawinyu** [56]	2022	Taiwan	22	34.1	20M/2 F	NO	26.3	6pp/16 w	Olecranon bone graft	NA	22	15.3	106.5°	92.8 %	90.6	5.3	10.9
**Lin** [57]	2023	Taiwan	AVN group:15	45.9	8M/7F	YES	7.8	4pp/11w	Distal Radius	NA	14	14.9	129.9°	77 %	81.3	11.9	20
			Non-AVN group:19	29.9	15M/4F	NO	8.7	3pp/16w			18	14.6	130.4°	95.4 %	80.7	9.6	13
**Bhat** [58]	2023	India	38	28.7	36M/2F	YES	4.6	5pp/19w/14d	Distal Radius	NA	38	15.7	NA	NA	NA	NA	NA
**Toosi** [13]	2023	Iran	CPGA + cell therapy:5	27.8	M	NO	48	1pp/4w	Mesenchymal stem cell (BM-MSC)	NA	4	NA	60.6 %	NA	NA	NA	NA
			Iliac crest bone graft:5	25.6			33.6	2pp/3w	Iliac crest		4		58 %				
**Burnier** [7]	2023	France	77	24	72M /5F	NO	34.9	40pp/35w	Distal Radius	NA	72	13.6	54.8°/54.7°	NA	NA	NA	NA
**Zondervan** [59]	2023	USA	8	20	M	YES	NA	pp	Iliac Crest	NA	6	12	143.7°	NA	NA	2.9	NA
**Delamarre** [60]	2024	France	60	27.5	59M/1F	6 AVN	19.7	20pp/40w	Distal Radius or Iliac crest	NA	51		71.1°/67.2°	83.2 %	85.4	NA	30

Table 2: AVN (Yes/No, if assessed in the study); Nonunion time: Time taken for nonunion of the scaphoid fracture in months; Site of scaphoid fracture: Specific location of the fracture in the scaphoid bone (pp: proximal pole, w: waist, d: Distal); Operation time: Duration of the surgical procedure in minutes; Number of healed: Number of successfully healed fractures; Time to Healing: Time taken for the fractures to heal in weeks; ROM (F/E): Range of motion in Flexion/Extension (if in percentage, it is compared to the opposite hand; if presented separately, it is shown as flexion and extension; if a single number, it represents the total wrist motion); Grip Strength: Measurement of the grip strength of the hand (if in percentage, it is compared to the opposite hand; if in numeric form, it is in kilograms); MMWS: Modified Mayo Wrist Score; DASH: Disabilities of the Arm, Shoulder, and Hand score; VAS: Visual Analog Scale for pain measurement.

### 3.3 Studies evaluating VBGs outcomes

Generally, 18 articles were identified using VBGs for scaphoid fracture non-union treatment (Table 3). A total of 427 patients were examined, with gender information available for 415 individuals. Among these, 361 were male (86.99 %) and 54 were female (13.01 %). The mean age of the patients was 28.7 years. Additionally, the average duration of nonunion was 26.8 months. Regarding the fracture locations, 213 fractures (60.34 %) were located at the proximal pole, 133 fractures (37.68 %) at the waist, and 7 fractures (1.98 %) at the Distal end. Among the included studies of this group, most of the studies used Distal Radius graft while limited numbers (2 studies) used other types of VBGs such as thump metacarpal [11] and medial femoral condyle [10]. Table 4 includes the characteristics and retrieved data from all studies which used VBG for scaphoid non-union treatment. Among them, 14 studies reported the mean time to healing as their outcomes evaluations. Random effects model analysis for pooled values of the mean time to healing through all evaluated studies of VBG group was 13.84 ± 3.12sd weeks. Ten studies have reported the grip strength values based on the percent of the contralateral wrist strengths as functional outcomes evaluation. Our pooled analyses of the extracted data including mean percent of the contralateral wrist grip strengths demonstrated an overall mean of 83.2 % ± 11sd through all 276 patients of the 10 studies. In addition, a total of 6 studies have evaluated and reported the MMWS of the VBG treated patients. The pooled mean value of this score of 195 patients through the studies was 86.2 ± 9.15sd. Regarding VAS and DASH scores evaluations, it should be considered that only 3 studies reported results of these outcomes measurements in VBG treated group.

**Table 4:** Characteristics of VBG studies.

Author	Year	Country	Sample size	Mean age	Sex M/F	AVN	Nonunion time	Cite of scaphoid fracture	Graft source	Operation time	Number of healed	Time to Healing	ROM (F/E)	Grip Strength	MMWS	DASH	VAS
Malizos [61]	2001	UK	22	30	22M	No	48	22pp	Distal Radius	NA	22	10	87°/38	85 %	NA	NA	NA
Steinman [62]	2002	USA	14	28	14M	4 AVN	34.1	8pp/6w	Distal Radius	NA	14	11	54°/48°	NA	NA	NA	NA
Sawaizumi [63]	2003	Japan	7	23	M 7M	No	9	2pp/5w	Vascularized second metacarpal-base bone	NA	7	10.4	60°/58.7°	NA	NA	NA	NA
Bartelli [11]	2004	Brazil	24	34	M 24M	2 AVN	96	14pp/8w	Thump metacarpal	NA	21	48	44.4°/39.3°	28.5 Kg	NA	NA	18
Chen [30]	2006	Taiwan	11	32	s 8M/3F	Yes	12.6	1pp/8w/1d	Distal Radius	NA	11	13.3	71°/85°	98.4 %	86.8	NA	NA
Jones [10]	2008	Austria	Distal radial pedicle vascularized graft:10	31	7M/3F	Yes	26	22w	Distal Radius	NA	4	19	NA	68 %	NA	NA	NA
			Vascularized medial femoral condyle graft:12	23	M		31		Medial femoral condyle		12	13		86 %			
Kapoor [64]	2008	UK	34	27	31M/3F	Yes	14	18pp/16w	Distal Radius	NA	NA	19.2	NA	NA	NA	NA	NA
Hamdi [65]	2011	Tunisia	26	32	23M/3F	1 AVN	17	NA	Distal Radius	NA	23	12	NA	NA	NA	NA	NA
Liang [66]	2013	China	11	28	10M/1F	Yes	28.8	11pp	Distal Radius	NA	11	11.4	129.4°	90 %	85	NA	NA
Rahimnia [67]	2018	Iran	41	26.7	35M/6F	Yes	47	33pp	Distal Radius	100	30	NA	NA	70 %	78	26	NA
Korompilias [68]	2019	Greece	11	28.6	10M/1F	No	NA	3pp/6w/2d	Distal Radius	NA	11	11.2	NA	NA	NA	4.7	NA
Tsumura [69]	2020	Japan	19	33	18M/1F	No	12.1	2pp/16w/1d	Distal Radius	NA	18	24	NA	NA	NA	NA	NA
Vakalopoulos [70]	2020	Switzerland	9	22.5	8M/1F	1 AVN	6	1pp/8w	Distal Radius	NA	9	16	NA	NA	NA	NA	NA
Papatheodorou [71]	2021	USA	64	27	45M/19F	Yes	23	27pp	Distal Radius	NA	55	12	117°	83 %	86	NA	NA
Shen [72]	2021	China	29	27	27M/2F	Yes	23	5pp/21w/3d	Distal Radius	NA	29	NA	132°	90 %	92	1	2
Siddiqui [73]	2021	Pakistan	25	38.2	19M/6 F	Yes	15.2	8pp/17w	Distal Radius	NA	25	12.4	58.4°/60.2°	95.8 %	NA	NA	20.3
Polat [74]	2022	Turkey	Femoral condyle group:18	31	34M/5F	9 AVN	24	39pp	Medial femoral condyle	221	17	13	110°	86 %	90	11	14
			Distal Radius gropu:21			11 AVN	17		Distal Radius	100	19	15	111°	90 %	90	11	20
Bugeja [75]	2023	UK	1,2-ICSRA group:8	29.6	19M	No	NA	19pp	Distal Radius	NA	8	NA	NA	NA	NA	NA	NA
			2,3-ICSRA group:11	22.5					Distal Radius		9						

AVN (Yes/No, if assessed in the study); Nonunion time: Time taken for nonunion of the scaphoid fracture in months; Site of scaphoid fracture: Specific location of the fracture in the scaphoid bone (pp: proximal pole, w: waist, d: Distal); Operation time: Duration of the surgical procedure in minutes; Number of healed: Number of successfully healed fractures; Time to Healing: Time taken for the fractures to heal in weeks; ROM (F/E): Range of motion in Flexion/Extension (if in percentage, it is compared to the opposite hand; if presented separately, it is shown as flexion and extension; if a single number, it represents the total wrist motion); Grip Strength: Measurement of the grip strength of the hand (if in percentage, it is compared to the opposite hand; if in numeric form, it is in kilograms); MMWS: Modified Mayo Wrist Score; DASH: Disabilities of the Arm, Shoulder, and Hand score; VAS: Visual Analog Scale for pain measurement.

### 3.4 Quality evaluation and risk of bias assessments results

**Fig. 2:**
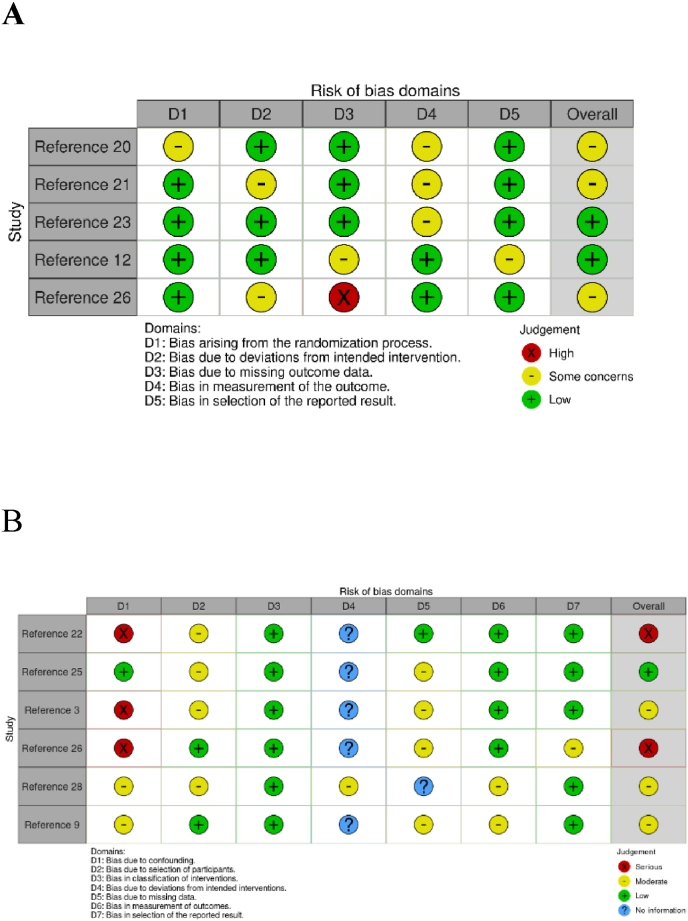
Risk of bias assessments Rob2 and ROBINS results of the A) Randomised Control Trials and B) Non-Randomised Comparative studies.

Overall ‘high risk’ were identified in three articles in comparative group, mostly due to the nature of being retrospective studies. In addition, the heterogeneous nature of the patient characteristics, treatments, comparators and outcomes of the interventions was reflected in their risk of bias Fig. 2. There have been also five studies with ‘high risk’ of bias amongst the NVBG group (Fig. S1) as well as no ‘high risk’ studies detected through the VBG group (Fig. S2). The certainty of evidence was assessed using GRADE (Table S1). Results demonstrated Moderate score for union rates and Grip, Low for time to union, ROM, and MMWS, reflecting bias and reporting challenges despite RCT contributions.

## 4 Meta analyses

### 4.1 Healing rate

As it was declared, we identified 10 studies which compared the outcomes of VBG vs NVBG for scaphoid non-union treatment. The number of total patients and healed ones were extracted from each study to analyse the healing rates. Based on the results of the forest plots obtained, it is known that the VBG can be more effective than the NVBG in terms of Healing rate scores in the scaphoid non-union after graft implantation. Statistically significant difference in treatment effect between the two groups was detected (Test for overall effect: Z:3.04, RR: 0.47; 95 % CI 0.30–0.73, p = 0.0007). A specific forest plot of these results is shown in Fig. 3A.

**Fig. 3A:**
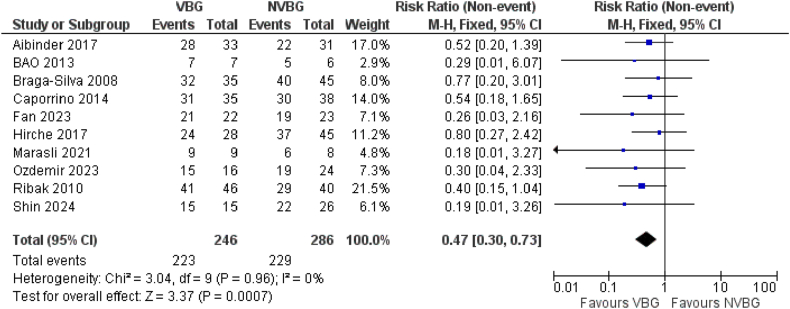
Healing Rate of Comparative studies.

For non-comparative NVBG (27 studies, n = 946) and VBG (16 studies, n = 345) cohorts, pooled union rates using random-effects models were calculated. The results, showed union rates of 92 % (95 % CI [85–91]) for NVBGs and 96 % (95 % CI [88–95]) for VBGs (Figs. S3 and S4). However, these analyses cannot be emphasized in the interpretations due to suspected reporting biases in number of fully healed cases, favouring higher healing rates specifically in VBGs.

### 4.2 Time to union

Data on the means ± standard deviations (SDs) of the time to union of scaphoid non-union after graft implantation, along with the sample sizes for each study group, were used to evaluate the pooled time to union in weeks for the comparative, NVBG, and VBG groups. The results for the comparative group demonstrated a statistically significant shorter time to union for patients who received VBG compared to those who received NVBG. Statistically significant difference in treatment effect between the two groups was detected. Fig. 3B illustrates the forest plot of this evaluation, where the random effects model shows the test for overall effect: Z = 14.58, 95 % CI [0.99–1.3], p < 0.00001. In addition, for the two other groups of studies that only reported the outcomes of scaphoid non-union implantation using VBGs or NVBGs separately, we calculated the random effects model to determine the integrated time to union in each group. Among the studies which reported the mean time to union, the integrated mean time to union for the VBG group was 13.84 ± 7.4 weeks (Fig. S5), while the integrated value for this outcome in the NVBG group was 14.80 ± 7.5 weeks (Fig. S6), seemingly a longer time.

**Fig. 3B:**
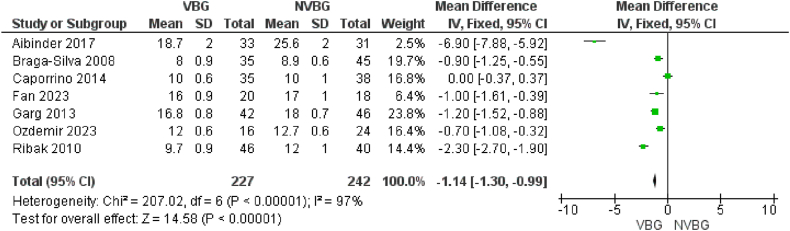
Time to Union of Comparative studies.

### 4.3 Range of motion (ROM)

The wrist flexion−extension ROM were extracted and calculated as Total ROM (flexion + extension) for each study. The meta analyses through comparative group significantly showed higher ROM in VBG treated patients compared to NVBG treated cases (Test for overall effect: Z = 4.95 (P < 0.0001)) (Fig. 3C). Regarding the pooled total ROM of VBG group and NVBG group using Random Effect Model, we also noted an slightly higher total ROM in VBG group (Figs. S7 and S8).

**Fig. 3C:**
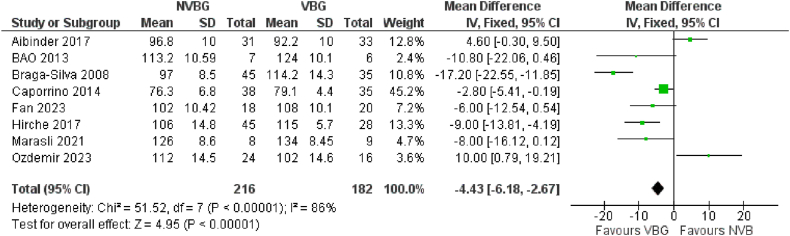
Total ROM of Comparative studies.

### 4.4 MMWS

A total of 5 studies in the comparative group, 17 studies in the NVBG group, and 6 studies in the VBG group reported the MMWS as the functional outcome of scaphoid non-union grafting. The mean values of these measurements were retrieved from the studies. The random effects model analysis of the comparative group demonstrated that the MMWS in patients who received VBG was significantly higher than the reported values in patients treated with NVBG. The overall effect (Z) was 3.18 with P = 0.001, 95 % CI [1.58–6.65]. Fig. 3D presents a forest plot illustrating a significant effect favoring VBG compared to NVBG. Additionally, we calculated the integrated overall mean values of MMWS for the VBG and NVBG groups separately. The results indicate a notably higher mean MMWS in the VBG group (86.28 ± 9.5 SD) (Fig. S9).

**Fig. 3D:**
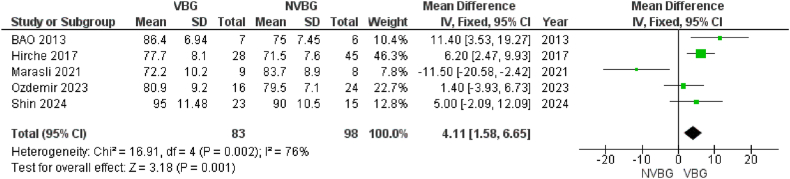
Total MMWS of Comparative studies.

### 4.5 Grip strength

A total of 7 studies in the comparative group, 24 studies in the NVBG group, and 10 studies in the VBG group reported the Grip strength based on the percent of the contralateral hand score as the functional outcome of scaphoid non-union grafting. The mean values of these measurements were retrieved from the studies. The random effects model analysis of the comparative group demonstrated that the Grip strength in patients who received VBG was significantly higher than the reported values in patients treated with NVBG. The overall effect (Z) was 3.53 with P = 0.0004, 95 % CI [1.68–5.88]. Fig. 3E presents a comparative forest plot illustrating a significant effect favoring VBG compared to NVBG regarding Grip strength. Additionally, we calculated the integrated overall mean values of Grip strength for the VBG and NVBG groups separately. The results indicate a mean value of Grip strength % of contralateral hand of (83.12 ± 14.25 SD) (Fig. S11) in the VBG group while the NVBG group had not a significantly different mean value of Grip strength (83.96 ± 11.6 SD) (Fig. S12).

**Fig. 3E:**
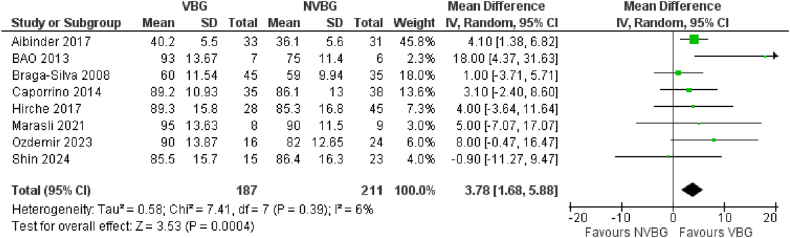
Grip Strength (% of contralateral wrist) of Comparative studies.

### 4.6 AVN vs Non-AVN

Next, we categorized the studies of each graft type and their outcomes based on the presence of avascular necrosis of the scaphoid (AVN). As subgrouping regarding the AVN status in comparative studies was not applicable due to not enough data; differences in time to union, grip strength, and MMWS between AVN and non-AVN studies for NVBG and VBG groups were analyzed using Welch's t-tests. Table S2 presents all the subgroup results. In NVBG group, non-AVN cases had shorter time to healing (4.73 weeks, p < 0.0001), better grip strength (6.09 %, p = 0.001) and MMWS (7.16, p < 0.0001) than AVN cases. For VBG, non-AVN cases showed higher MMWS (4.90, p = 0.016), with no significant differences in grip strength (2.65 %, p = 0.065) or time to healing (0.42 weeks, p = 0.374). In summary, we can infer from the table that scaphoids without AVN treated with NVBG show some functional advantages. Additionally, patients with scaphoid AVN who received NVBG treatment recorded the worst time to healing scores as mentioned in Table S2. Other hands, VBGs showed a higher MMWS SCORE in non-AVN but no clear differences in healing or grip strength. If we ask what kind of grafts (VBGs or NVBGs) show better outcomes in AVN patients, we can refer to Table S3, where the comparisons results suggest that applying VBGs in AVN cases can lead to shorter time to healing and higher MMWS and Grip scores. These results must be noted cautiously, as the AVN status in each group was reported by small sample sizes and other comparisons were not applicable due to the low number of reported outcomes for some subgroups highlighting research gaps needing further investigations.

### 4.7 Bone biomaterial grafts

Application of bone biomaterials for the treatment of bone fractures, particularly nonunions represents a promising modality in hand orthopedic surgery [12]. Synthetic or natural bone biomaterials offer several advantages over the traditional autografts and allografts, including the elimination of donor site morbidity, consistent quality, and the potential for customization to meet specific clinical needs. In this systematic review, we found one RCT article (Toosi et al.) which compared the outcomes of application of a collagen/polyglycolic acid (CPGA) biomaterial scaffold enriched with bone marrow mesenchymal stromal cell (BM-MSC) versus iliac crest bone graft therapy in scaphoid non-union patients. Their findings were particularly noteworthy, demonstrating same healing outcomes that were intriguingly comparable to those achieved with iliac crest NVBG [13]. They interestingly reported that restorative effects of CPGA + cell therapy were similar to those of NVBG standard therapy, except for the grip strength (*P* = 0.048) and QDASH score (*P* = 0.044) changes, which were higher in the CPGA + cell therapy group. Three months following the surgery, radiographic images and computed tomography (CT) scans also demonstrated that the scaphoid union rate in the bone biomaterial received group was comparable to the group treated with the standard NVBG. This opens up a new window in the field of scaphoid non-union treatment and suggests that developed bone biomaterials can potentially meet the efficacy of traditional grafts in promoting bone healing and functional recovery. Despite the promising results reported by Toosi et al., the available evidence base for synthetic biomaterials in scaphoid fracture treatment remains limited. This sole study (n = 10 patients), precluded robust meta-analysis for this category of bone grafts, and we interpret these findings as preliminary. There is a pressing need for large-scale, randomized controlled trials to fully evaluate the safety and efficacy of these synthetic biomaterials. Future research should focus on the assessments of the long-term outcomes in which the durability and integration of bone biomaterial grafts over the extended period of time. In addition, optimizing the best formulations and combinations of bioactive agents to maximize bone healing is substantial. Finally performing further superiority/non-inferiority studies in which synthetic or natural polymeric bone biomaterials with both NVBGs and VBGs under the same clinical conditions get compared to validate their effectiveness is necessary.

## 5 Discussion

A scaphoid fracture is a critical injury that often needs surgical treatment due to its importance in the human activity [76]. However, even after successful surgery, there is a chance that leads to nonunion. In this situation, the surgeon needs to decide based on the circumstances of the fractured scaphoid including the vascularity status, the site of the fracture or the availability of the donor bone resources, choose one of the possible grafts options for implantation into the defect site [1]. This systematic review and meta-analysis provides valuable insights into the effectiveness of different grafting techniques for treating scaphoid fractures, particularly those complicated by nonunion and avascular necrosis (AVN). The majority of findings in this study are consistent with prior meta-analyses, which have demonstrated superior union rates, improved range of motion, and favorable clinical outcomes with VBGs. For instance, our results align with the observations of Zhang et al. [6], who similarly reported enhanced union rates and reduced time to union with VBGs. Additionally, while our findings corroborate those of Duncombe et al. regarding the efficacy of VBGs, our more stringent inclusion criteria—prioritizing higher methodological quality and more recent studies—yielded a more refined and contemporary evidence base. By comparing NVBGs, VBGs, and bringing up the possibility of newly immerged bone biomaterial grafts, our results demonstrate significant differences in clinical outcomes through comparative studies, with VBGs showing higher union rates and shorter healing times than NVBGs, though pooled analyses of separate studies showed less pronounced differences due to overlapping confidence intervals. This evidence-based guidance is crucial for orthopedic surgeons. Meanwhile, we should pay attention to the detected ROBs through different categories of studies included in the meta-analyses. The RoB in 3 comparative studies, may potentially overestimate some treatment effects like union rates. Additionally, 5 NVBG studies (Fig. S2) with high risks, may inflate outcomes such as healing times or functional scores due to selection bias or lack of blinding. Besides, no high-RoB studies were detected in the VBG group (Fig. S1). This distribution suggests that NVBG outcomes might be more biased upward, reducing overall certainty in comparative efficacy, as reflected in the GRADE ratings (Table S1). While a number of previous systematic reviews have addressed some extents of this subject [1,77,78]; the strength of our study lies in its inclusion of newly published articles from 2022, 2023, and 2024. To our knowledge, as of April 2024, no prior reviews have comprehensively evaluated, categorized, and meta-analyzed all published papers from this period, including cohort studies, case series, and randomized controlled trials (RCTs). Furthermore, we provide additional insights by comparing the pooled effectiveness of each type of graft in AVN and non-AVN scaphoids, which were not considered in previous systematic reviews. In this study, data from 2332 scaphoid fracture patients who received VBG, NVBG, or bone biomaterials were extracted, categorized, and meta-analyzed. Moreover, this study compares and discusses the outcomes of new bone graft options, such as synthetic or natural polymeric bone biomaterials, with the other mentioned grafts, opening up new possibilities for the treatment of scaphoid nonunion.

### 5.1 Vascularized bone grafts (VBGs)

The outcomes favoring VBGs in terms of healing rates and faster union times are particularly considerable. Although several published studies in the literature have favored this type of grafts for scaphoid nonunions, some others have reached conflicting conclusions in some outcomes such as time to union [10,23,28,69], total ROM [11,28,62,74], MMWS [26,67] and the grip strength [10,11,67] see Fig. 3B–S6, 3C, S8, 3D, S11, 3E and S13. The fact of VBGs ability to provide a sustained blood supply to the grafted tissue offers a distinct biological advantage over NVBGs, especially in scaphoid fractures where AVN is a major contributing factor to nonunion. The patients with AVN in scaphoid, which are totally difficult to treat due to their limited blood supply to proximal parts of the scaphoid, showed marked improvement with VBGs shorter time to healing [66,71], higher grip strengths [66,72,73]. This confirms the biomechanical and biological rationale for using VBGs, which supply the necessary osteogenic and osteoconductive environment for robust healing. Some comparative studies have compared the efficacy of VBGs from different sources such as medial femoral condyle and Distal Radius [10,74], with challenging conclusions. However most of the studies have used VBG from Distal Radius and were included in the meta-analyses. Our findings align with previous studies, such as those performed by Pinder et al. (2015) [78], which similarly reported better outcomes with VBGs for nonunion of scaphoid fractures. Although general pooled evidence on the outcomes in separate groups indicates higher success rates for VBGs (earlier time to union, improved functionality scores, and greater total ROM postoperatively), the overlapped confidence intervals (e.g., ROM: VBG 122.58° [CI 109.71–135.45] vs. NVBG 119.35° [CI 105.74–132.96]) may preclude definitive superiority claims for VBGs. However, the findings of comparative analyses underscore the value of VBGs more evidently in the present study, particularly in cases with high complexity and compromised vascularization. Still, treatment decision-makers should consider clinical context and case-specific conditions regarding the use of these grafts. Further research is needed to confirm these trends.

### 5.2 Non-vascularized bone grafts (NVBGs)

Despite the clear advantages of VBGs, NVBGs remain a viable option, particularly in cases where vascularity is not severely impaired, and the AVN is not detected. The results show that NVBGs, typically harvested from the iliac crest or Distal Radius, offer simplicity and effectiveness for many patients specifically those without AVN [54,55]. However, their reliance on the surrounding bone's vascular bed for integration inherently limits their applicability in cases of proximal pole fractures or where avascular necrosis is present although studies reported controversial results of its effectiveness [36,47]. Our meta-analysis demonstrated that while NVBGs can achieve high union rates, they require longer healing times compared to VBGs, and functional recovery, as measured by grip strength and MMWS, is also slightly diminished. On the other hand, treating patients with scaphoid AVN with NVBGs seems not as effective as VBGs as it is shown in Table S1. The results emphasize that NVBGs should be carefully selected based on the patient's specific fracture characteristics, as they may not be the ideal choice when vascularity is compromised.

### 5.3 Emerging potential of bone biomaterial grafts

The use of synthetic or natural polymeric bone biomaterial grafts in scaphoid fracture treatment represents a promising frontier. Although the evidence base for bone biomaterials grafts in scaphoid defects remains limited, the single included study performed by Toosi et al. (2023) [13] showed comparable and interesting outcomes between a bone biomaterial composed of collagen/poly glycolic acid (CPGA) scaffolds enriched with bone marrow mesenchymal stem cells (BM-MSCs) with traditional NVBGs from iliac crest. The incorporation of bioactive agents such as BMP-2 and VEGF into the applied bone scaffolds shows significant potential for enhancing osteogenesis and angiogenesis [79,80]. This single biomaterial based study provides preliminary evidence of efficacy comparable to NVBGs, but direct comparison with NVBGs and VBGs in a meta-analytic framework was not feasible due to insufficient data. In addition, its scarcity underscores the use of biomaterials significance as a pioneering approach that opens a new era in scaphoid nonunion management. This is particularly relevant in scenarios where autografts are unavailable or undesirable due to concerns regarding donor site morbidity, especially when promoting vascularity at the defect site is essential. However, they face challenges in cost, availability, and risks. These grafts may include higher costs and limited availability due to specialized production, contrasting with autografts' accessibility. Risks include immune reactions, and mechanical failure, and similar to NVBG's avascularity risks or VBG's surgical complexity. Future vascularized biomaterials may balance these factors, meriting further research. Despite these promising findings, there is a clear need for more large-scale randomized controlled trials (RCTs) to confirm the long-term efficacy and safety of various biomaterials in the treatment of scaphoid nonunions. Furthermore, future research should prioritize optimizing the composition and delivery of bioactive agents to fully harness the potential of synthetic grafts as substitutes for traditional autografts, particularly as alternatives to vascularized bone grafts (VBGs).

### 5.4 Clinical implications and future directions

This study highlights the critical need for a tailored approach to treating scaphoid nonunion, based on fracture characteristics and the availability of grafting materials. Complication rates associated with bone grafts for nonunion treatment highlight a critical trade-off between procedural risks and therapeutic efficacy. NVBGs, while simpler and widely utilized, often exhibit lower rates of union and almost one week delayed healing, as evidenced by our findings through comparative studies, potentially reflecting selective reporting of successful outcomes. Conversely, VBGs demonstrate better functional outcomes, such as higher grip strength and MMWS compared to NVBGs, yet they are associated with increased surgical complexity and risks, including donor site morbidity and vascular anastomosis failure and a heightened infection risk due to prolonged operative time and additional incisions. Despite these risks, VBGs’ ability to enhance vascularity may mitigate AVN progression, offering a compelling efficacy advantage, particularly in complicated cases. This means VBGs should be the preferred option in where vascularity is compromised or AVN is detected, while NVBGs may be sufficient in simpler, full or semi-vascularized scaphoids. Results of this study show VBGs trend toward better outcomes in AVN cases and NVBGs show shorter time to healing in non-AVNs, but need more evidence in future studies.

Bone biomaterials, although in their early stages, offer exciting possibilities for minimizing the morbidity of the source site associated with autograft harvesting. Further research into the long-term efficacy of these biomaterials as substitutes in the scaphoid nonunion defect sites is essential. In terms of future directions, large-scale RCTs comparing VBGs, NVBGs, and bone biomaterial grafts under similar clinical conditions will be crucial for further refining treatment protocols for scaphoid nonunion treatment. Additionally, comprehensive molecular and biomechanical studies exploring the exact mechanisms of VBG superiority and bone biomaterial grafts enhanced with bone regeneration power and vascularization possibility could yield insights that improve graft performance across the board.

This meta-analysis has several limitations. The heterogeneity of the included studies, variation in outcome measures, and the limited studies on bone biomaterials hinder the generalizability of the findings. Upon reviewing the included studies, we encountered several challenges and pitfalls related to the content and design of the studies, echoing concerns noted by Ferguson et al. [77]. We actively tried to reduce possible bias and heterogeneities among the included studies as much as possible. For example, we decided to include studies using usual screw fixation tools as a stabilizing technique for bone grafts, provided their primary focus was on VBG or NVBG outcomes (and not the fixation screws-centered studies or methods, specifically the studies with possible industrial intentions). As is common in clinical research, there exists a risk of reporting bias, as surgeons and trainees are often motivated to publish their results primarily when they achieve favorable outcomes. Conversely, there tends to be less enthusiasm from surgeons—and possibly editors—to publish results that do not match the positive outcomes reported in previously published clinical series.

Additionally, in many studies, the evaluation of radiographic union following bone grafting was not conducted by an independent observer and were not randomized. This lack of randomization and independent assessment may introduce bias, particularly in cases where the outcome is uncertain, potentially leading to an inclination to report a successful outcome. It is important to note that many acute scaphoid fractures may initially show partial union across only part of their cross-section, making the fracture still visible [5], yet still classified as non-united. These partial unions typically progress to full consolidation over time. If such cases are mistakenly categorized as 'non-unions' and treated surgically, they can artificially inflate the reported success rates of bone graft procedures. The concern lies in the distinct molecular and pathophysiological characteristics of true non-unions, which impair the healing process in certain patients. If fractures that are partially united—or likely to unite with additional time—are erroneously categorized as non-unions and included in the analysis, this could artificially elevate success rates. It should be noted that inconsistent reporting across studies and variability in reported fracture locations precluded subgroup analysis by fracture site. This limitation highlights the need for standardized reporting systems to enable specific evaluations in future research. Additionally, these potential publication biases and the absence of standardized reporting across studies may have influenced the overall results. Future studies should aim for more standardized outcome measures and larger, well-designed trials to address these limitations.

## 6 Conclusion

This systematic review and meta-analysis comprehensively compared the effectiveness of non-vascularized bone grafts (NVBGs), vascularized bone grafts (VBGs), and bone biomaterial grafts in treating scaphoid fractures with nonunion. The results of comparative studies show that VBGs can offer higher union rates, faster healing times, and better functional outcomes, particularly in complex cases with compromised blood supply, such as scaphoids with avascular necrosis (AVN) though evidence certainty is moderate and clinical differences may be modest. NVBGs are effective in simpler cases whiteout AVN and may not have appropriate performances in AVN cases. Synthetic or natural polymeric bone biomaterials, though still emerging, show promise but need further research. These findings guide tailored treatment while highlighting the need for robust trials.

### 6.1 Research registration

Unfortunately, the review was not registered prospectively in a public registry such as PROSPERO. While the review adhered to PRISMA guidelines and followed a predefined search strategy (Table 1), the absence of prior registration may reduce transparency. To mitigate this, we have provided a detailed methodology and made all data available for scrutiny, but future reviews should prioritize prospective registration to enhance reproducibility and credibility.

## Informed consent statement

Not applicable.

## Author contributions

All authors have read and agreed to the published version of the manuscript.

## Data statement

All data generated or analyzed during this study are included in this published article (and its supplementary information files). Raw data are available from the corresponding author on reasonable request.

## Declaration of AI and AI-assisted technologies in the writing process

During the preparation of this work the authors used ChatGPT in order to improve readability and language of this study. After using this tool/service, the author(s) reviewed and edited the content as needed and take full responsibility for the content of the publication.

## Funding

No funds.

## Declaration of competing interest

The authors declare no conflict of interest.
