# Supplementary material for: Comparative efficacy of non-vascularized and vascularized bone grafts, with emerging insights into bone biomaterial grafts, in scaphoid fracture nonunion treatment: A systematic review and meta-analysis
Source: J Orthop Translat. 2025 Jun 28;53:231–45. doi: 10.1016/j.jot.2025.06.009 (PMC12269465; doi:10.1016/j.jot.2025.06.009)
Supplement: Multimedia component 1 [file mmc1.docx]

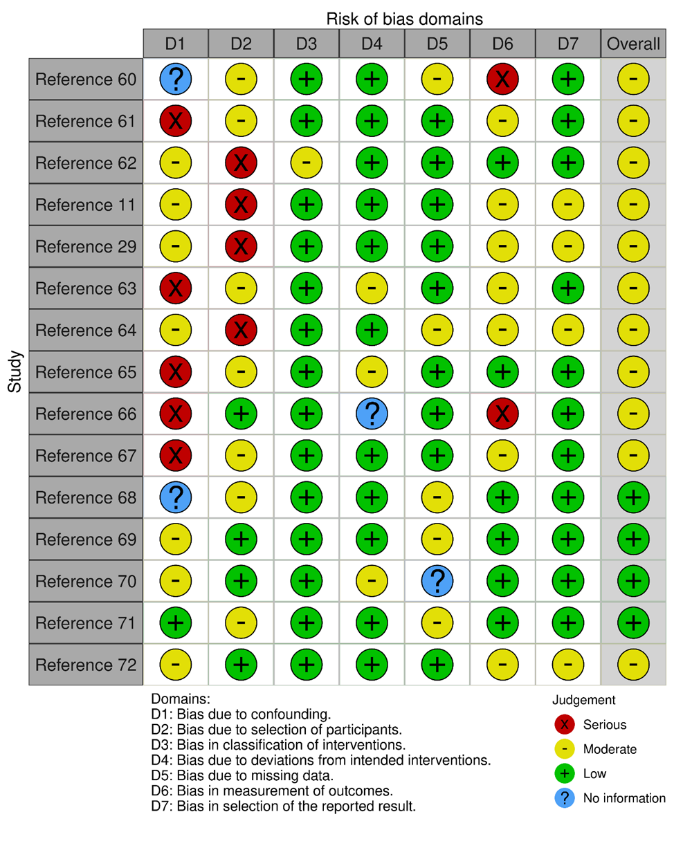


Figure S1: Risk of bias assessments result of VBG group


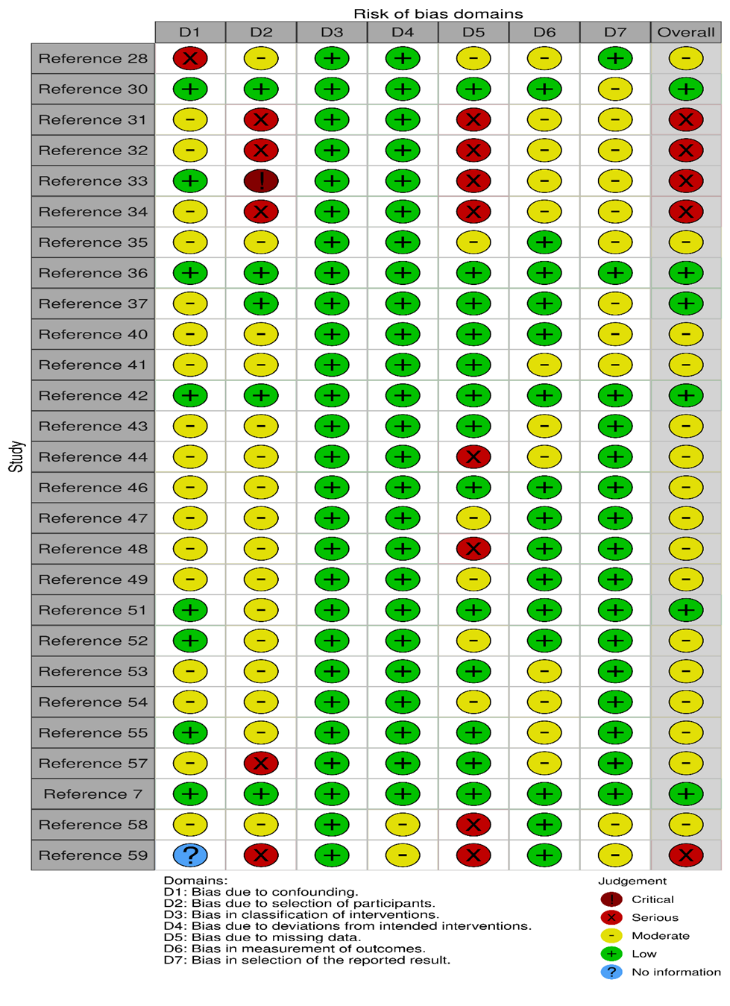


Figure S2: Risk of bias assessments result of NVBG group

**Table S1: GRADE Results**

| **Outcome** | **Studies (n)** | **Participants (n)** | **Effect Estimate** | **Certainty of Evidence** | **Reasons for Rating** |
| --- | --- | --- | --- | --- | --- |
| **Union Rates (NVBG vs. VBG)** | 10 | 452 | RR 0.50 [0.27–0.73] | Moderate | Started at Low due to predominantly observational design; downgraded 1 for publication bias (successful outcomes likely over-reported); upgraded 1 for large effect (RR 0.50). No downgrade for inconsistency (I² = 0%, moderate) or imprecision (narrow CI). |
| **Time to Union (NVBG vs. VBG)** | 7 | 382 | MD -1.13 wk [-1.31, -0.99] | Low | Started at Low due to predominantly observational design. Downgraded 1 for publication bias (shorter times likely over-reported) and 1 for inconsistency (I² > 70%, high). No indirectness, or imprecision (narrow CI) were detected. 1 upgrade (high effect, SMD ≈ -1.13). |
| **ROM (NVBG vs. VBG)** | 8 | 204 | MD ≈ 4.11° [-6.15-2.14] | Low | Started at Moderate due to balanced RCT (3) and observational (4) design; downgraded 1 for high RoB in 3 studies and 1 for publication bias (better outcomes likely over-reported). 1 downgrade for inconsistency (I² > 70%, moderate), indirectness, or imprecision (narrow CI). 1 upgrade (high effect, SMD ≈ 4.14). |
| **MMWS (NVBG vs. VBG)** | 5 | 181 | MD 4.11 [1.58-6.65] | Low | Started at High due to RCT majority (3/5); downgraded 1 for high RoB in 2 studies, and 1 for publication bias. 1 downgrade for inconsistency (I² > 75%, moderate). 1 upgrade indirectness (high effect, SMD ≈ 4). |
| Grip Strength (NVBG vs. VBG) | 8 | 384 | MD 3.78 [1.68, 5.88] | Moderate | Started at Moderate due to balanced RCT (4) and observational (3) design; No downgraded for RoB in 2 studies. 1 downgrade for publication bias. No downgrade for inconsistency (I² = 18%), indirectness, or imprecision (narrow CI). 1 upgrade (high effect, SMD ≈ 3.76). |

RR = Risk Ratio; MD = Mean Difference; wk = weeks; CI = Confidence Interval; RoB = Risk of Bias.


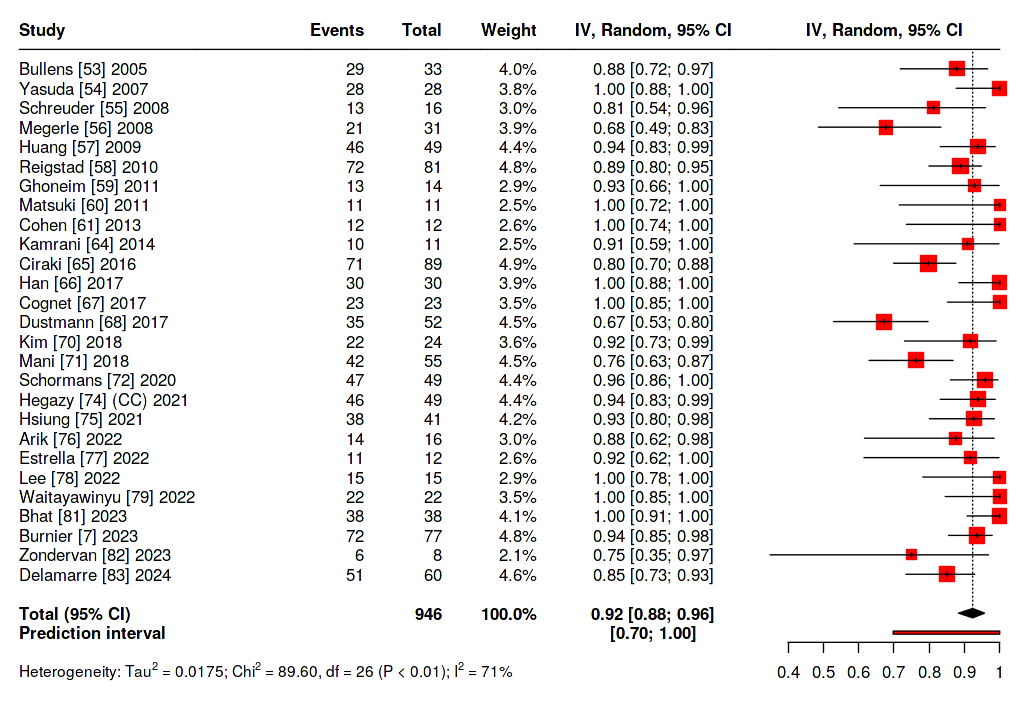


Figure S3: Healing Rate of NVBG studies


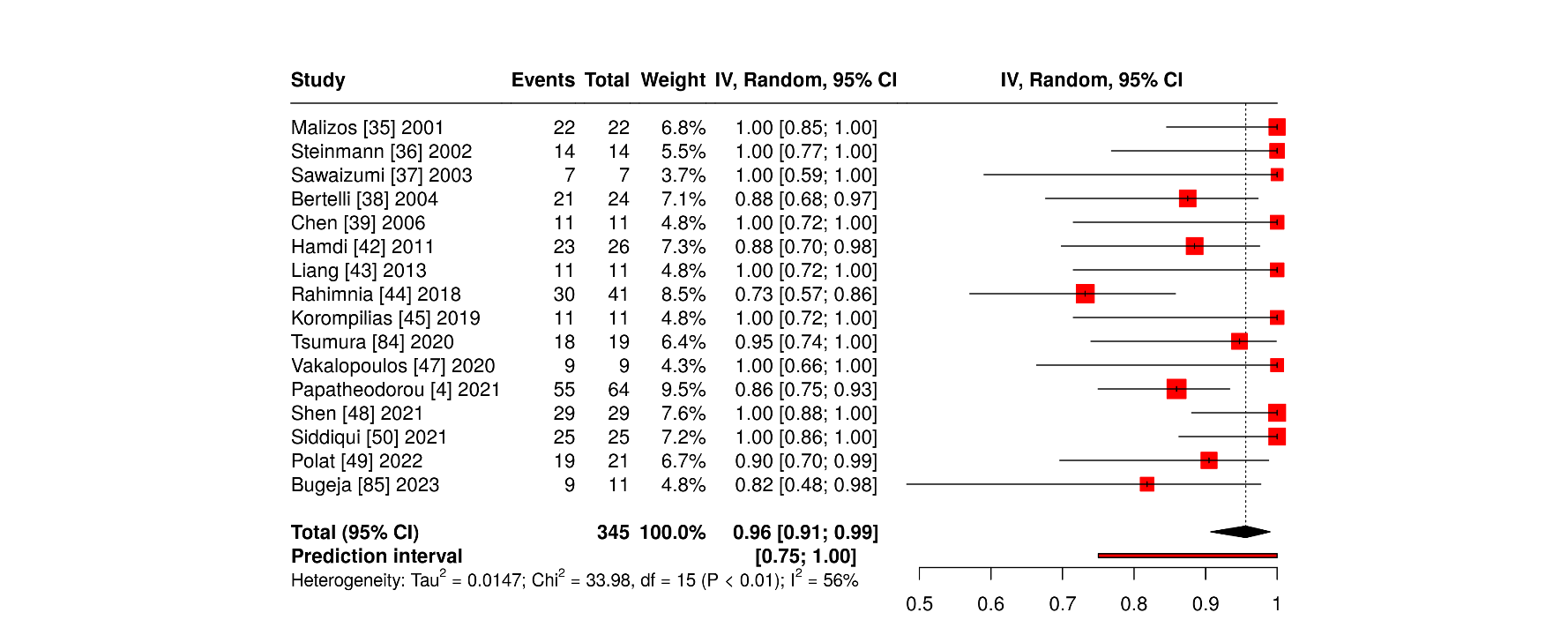


Figure S4: Healing Rate of VBG studies


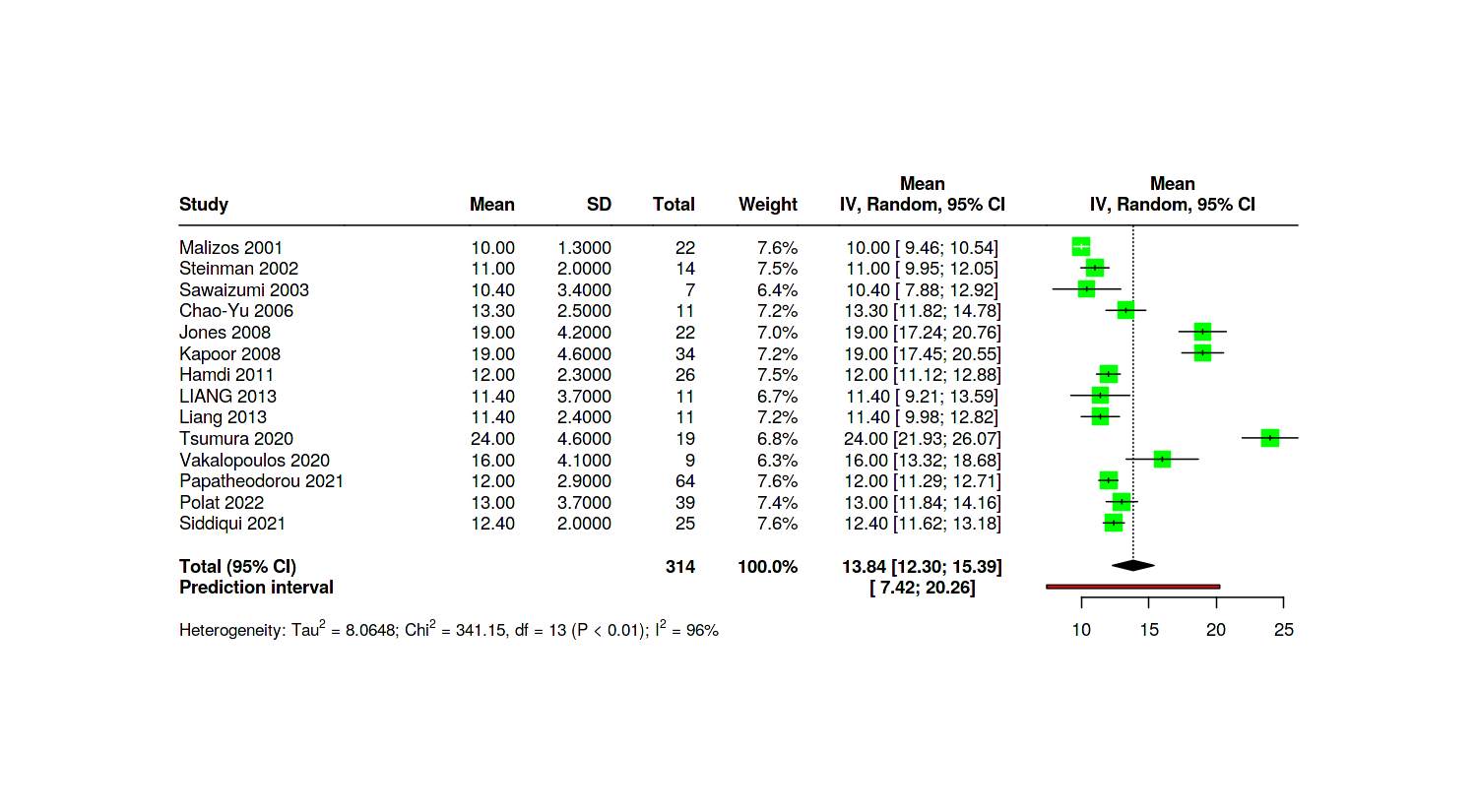


Figure S5: Time to union of VBG treated non-union scaphoids (weeks)


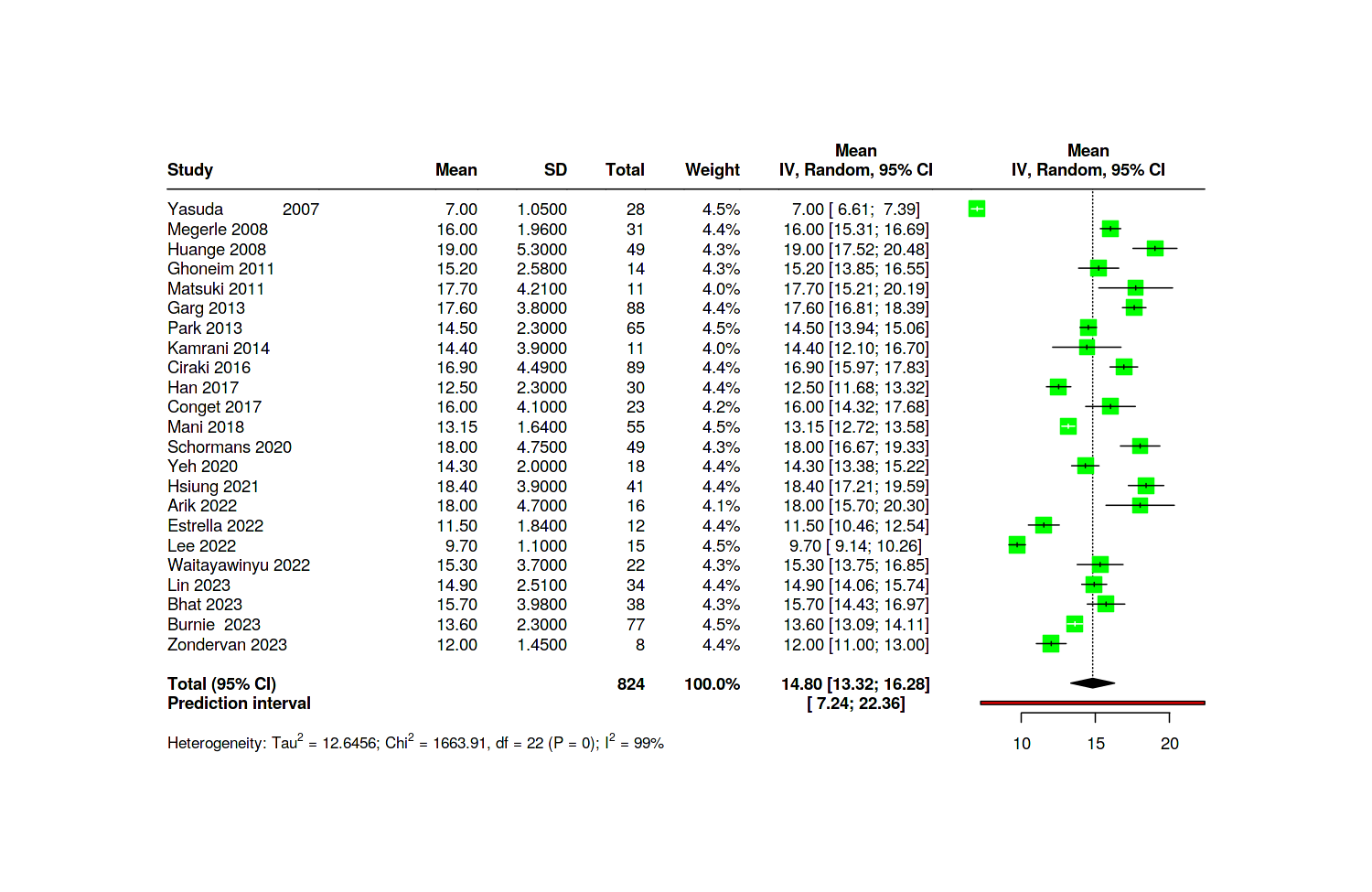


Figure S6: Time to union of NVBG treated non-union scaphoids (weeks)


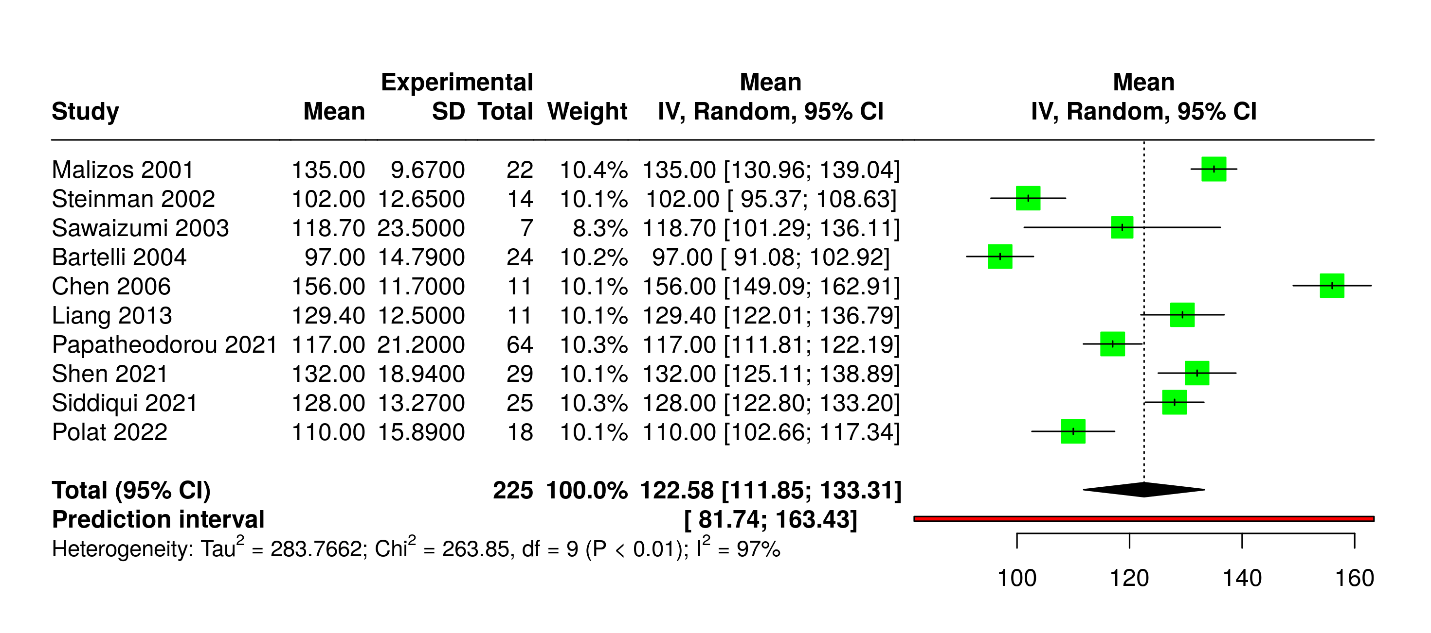


Figure S7: Total ROM of VBG treated non-union scaphoids


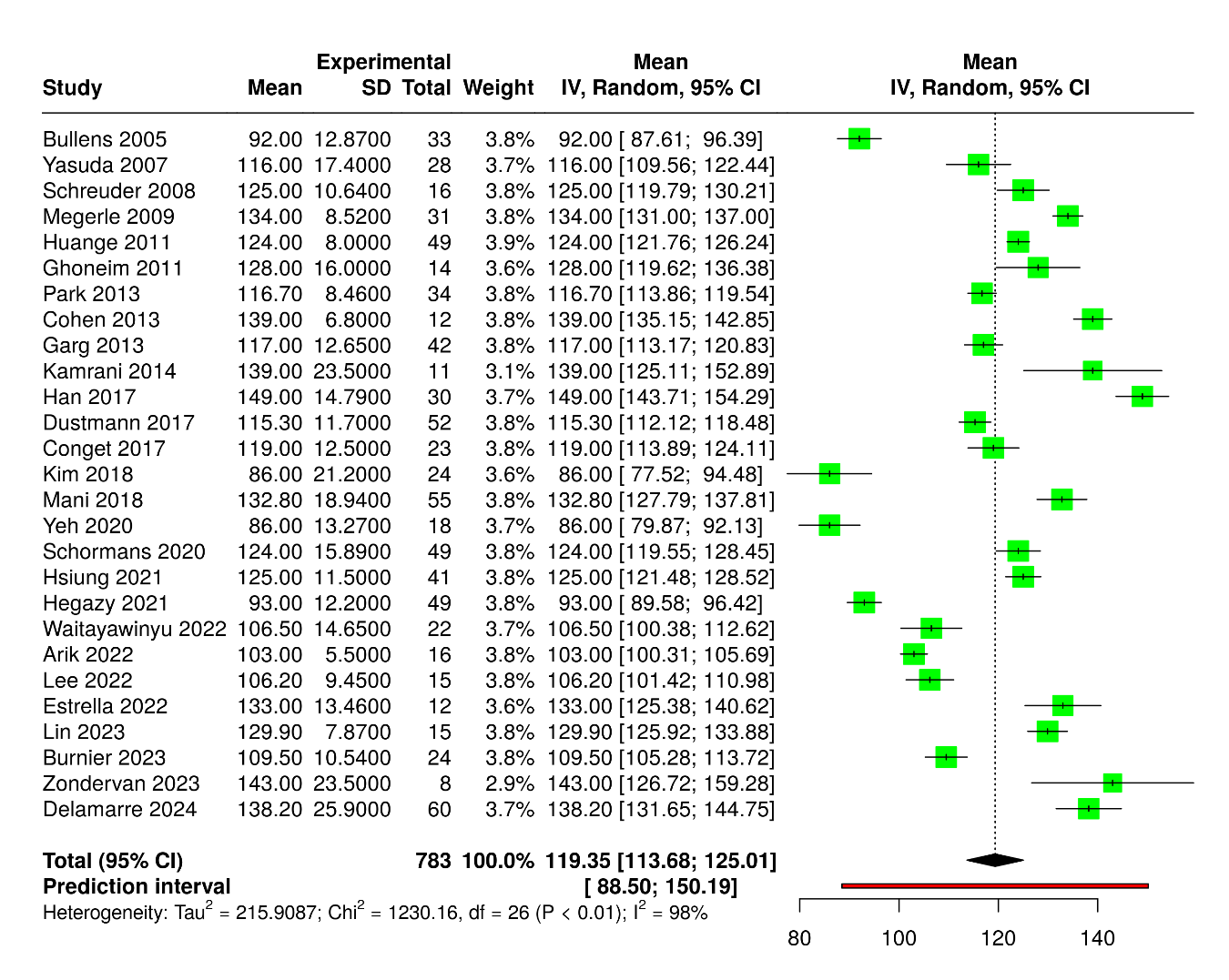


Figure S8: Total ROM of NVBG treated non-union scaphoids


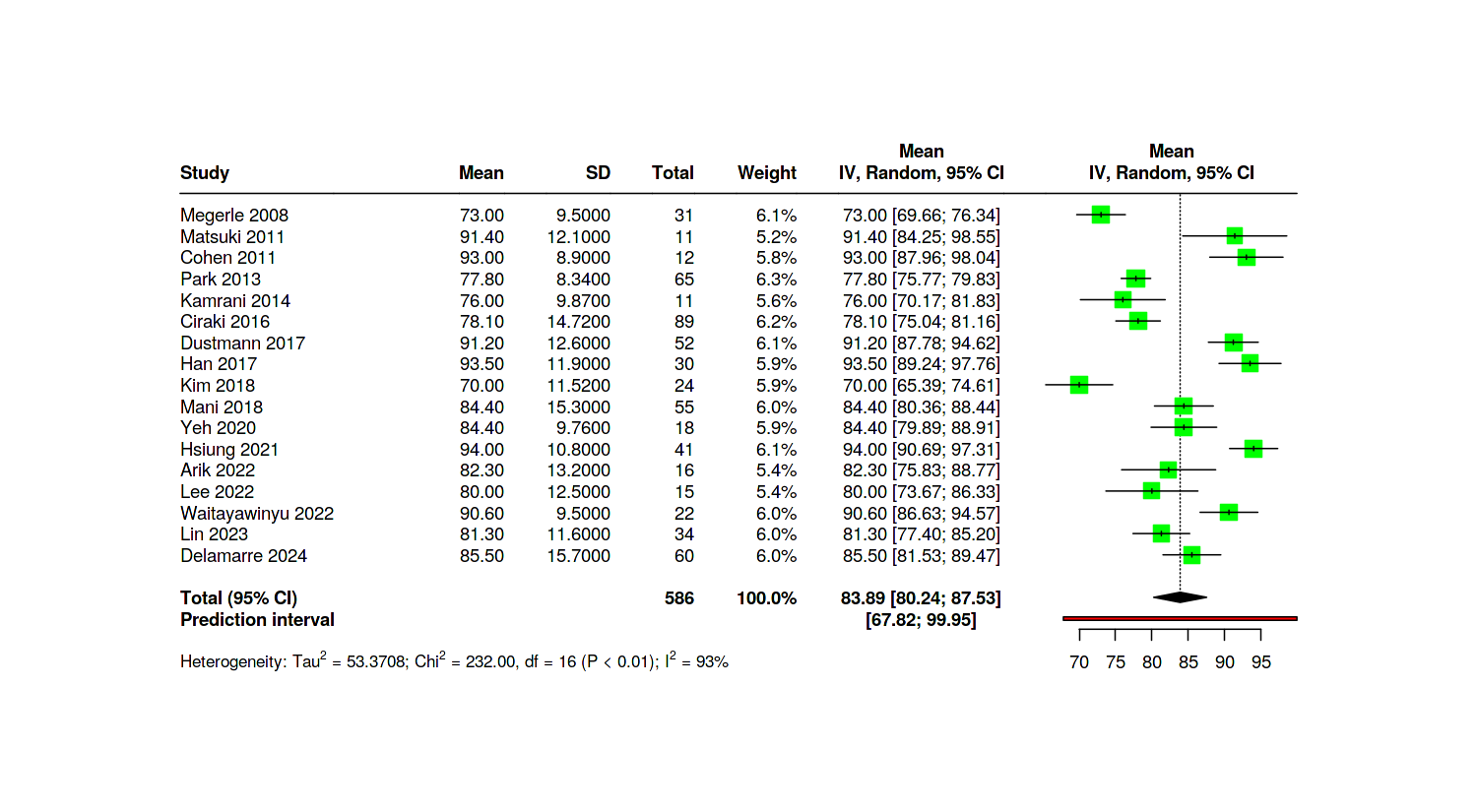


Figure S9: MMWS of NVBG treated non-union scaphoid


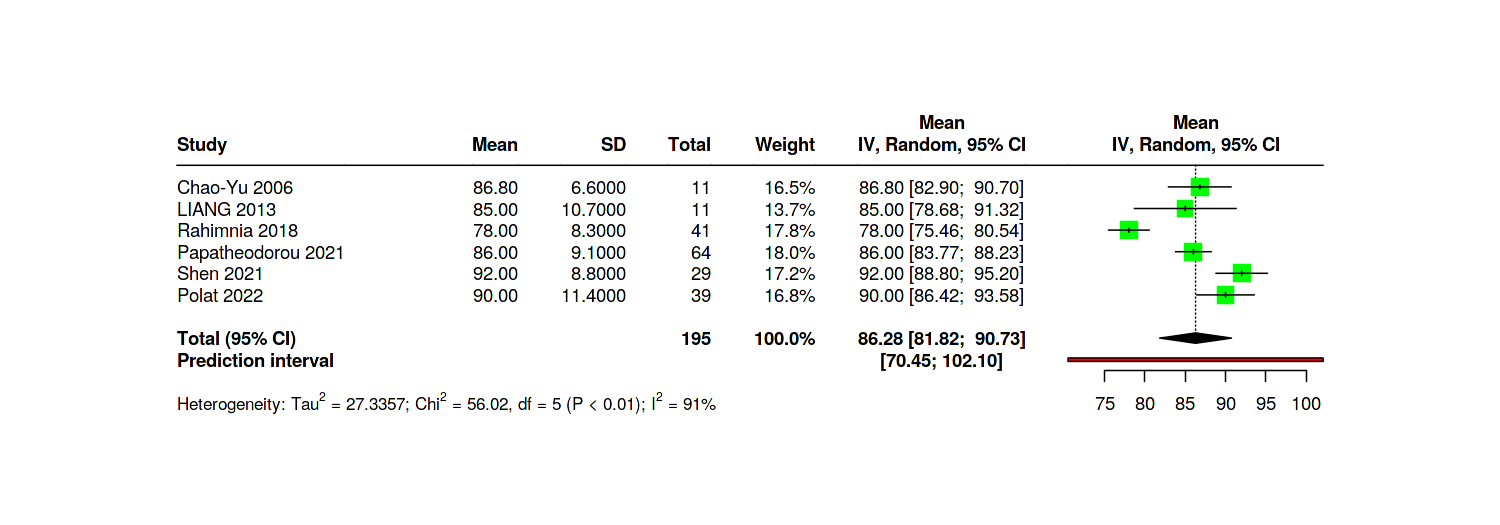
Figure S10: MMWS of VBG treated non-union scaphoid


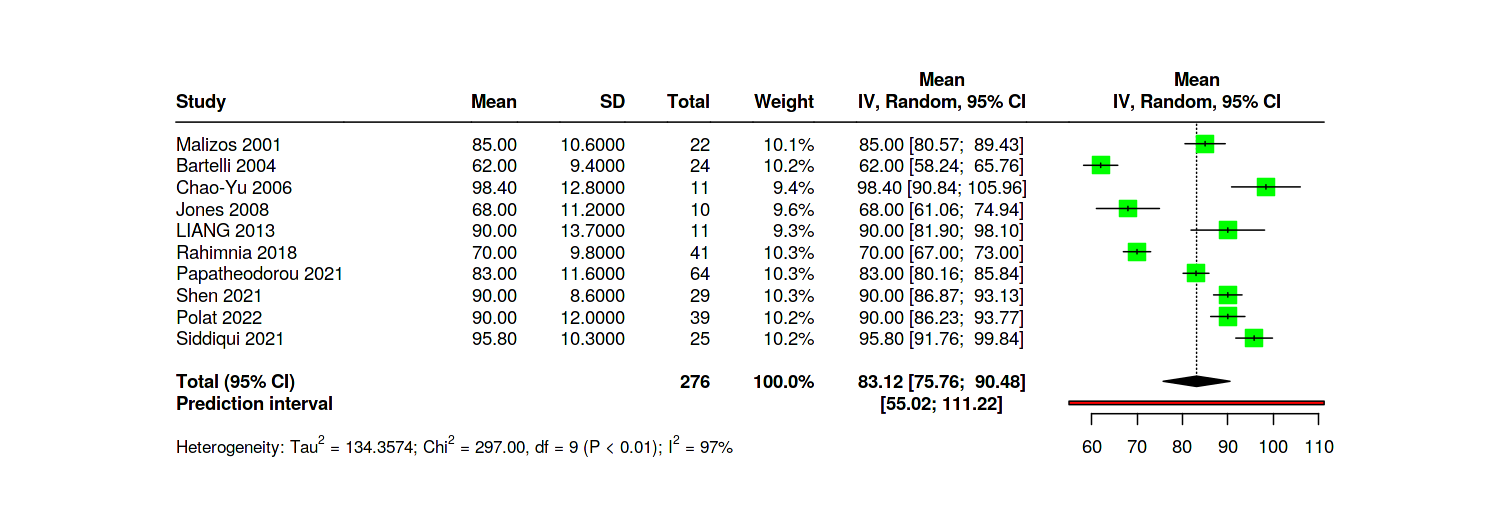


Figure S11: Grip Strength (% of contralateral wrist) of VBG studies


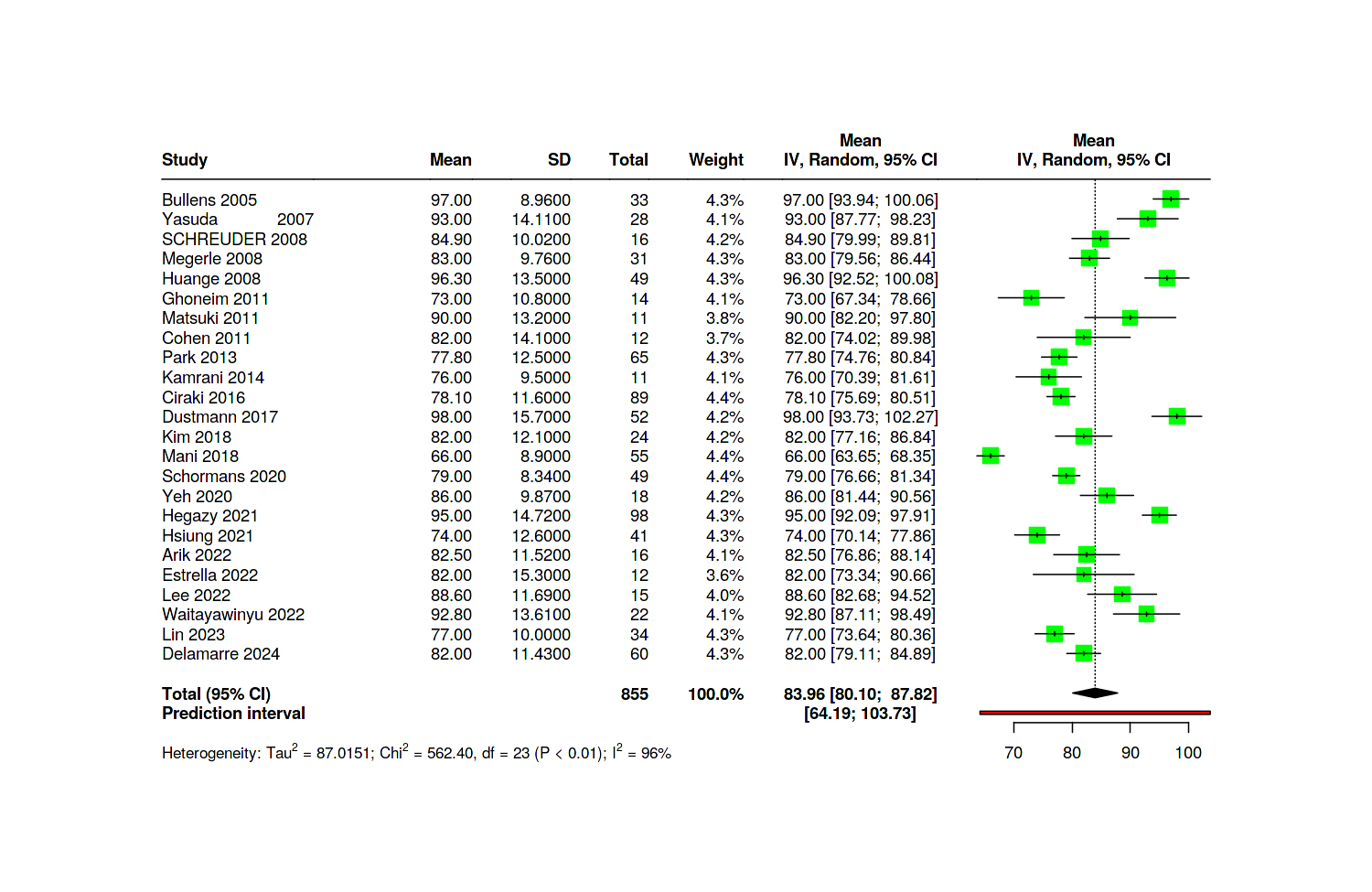


Figure S12: Grip Strength (% of contralateral wrist) of NVBG studies

Table S2: Comparison of Outcomes Between AVN and Non-AVN Studies for NVBG and VBG

| Graft Type | Outcome | AVN | Studies | N | Mean (SD) | Difference (95% CI) | p-value | Effect Size (d) |
| --- | --- | --- | --- | --- | --- | --- | --- | --- |
| NVBG | **Time to Healing (weeks)** | **(Yes)** | 7 | 17 | 17.57 (13.74) | 4.73 (3.703 to 5.757) | <0.0001 | 0.38 |
|  |  | **(No)** | 25 | 90 | 12.84 (3.41) |  |  |  |
|  | **Grip Strength (%)** | **(Yes)** | 4 | 65 | 78.91 (13.79) | 6.09 (2.625 to 9.555) | 0.001 | 0.49 |
|  |  | **(No)** | 19 | 72 | 85.00 (12.13) |  |  |  |
|  | **MMWS (score)** | **(Yes)** | 4 | 65 | 77.50 (12.21) | 7.16 (3.957 to 10.363) | <0.0001 | 0.55 |
|  |  | **(No)** | 14 | 84 | 84.66 (13.29) |  |  |  |
| VBG | **MMWS (score)** | **(Yes)** | 6 | 16 | 85.10 (9.41) | 4.90 (1.049 to 8.751) | 0.016 | 0.50 |
|  |  | **(No)** | 1 | 39 | 90.00 (11.40) |  |  |  |
|  | **Grip Strength (%)** | **(Yes)** | 7 | 19 | 83.45 (10.83) | 2.65 (-0.143 to 5.443) | 0.065 | 0.24 |
|  |  | **(No)** | 3 | 85 | 80.80 (10.97) |  |  |  |
|  | **Time to Healing (weeks)** | **(Yes)** | 4 | 13 | 14.94 (3.61) | 0.42 (-0.503 to 1.343) | 0.374 | 0.12 |
|  |  | **(No)** | 5 | 83 | 14.52 (3.19) |  |  |  |

Table S3: VBG vs. NVBG Outcomes in AVN Patients

| Outcome | VBG Mean (SD) | NVBG Mean (SD) | Difference (95% CI) | p-value | Effect Size (d) |
| --- | --- | --- | --- | --- | --- |
| Time to Union (weeks) | 14.94 (3.61) | 17.57 (13.74) | 2.63 (1.300 to 2.900) | <0.0001 | 0.60 |
| Grip Strength (%) | 83.45 (10.83) | 78.91 (13.79) | 4.54 (0.853 to 8.227) | 0.018 | 0.39 |
| MMWS (score) | 85.10 (9.41) | 77.50 (12.21) | 7.60 (4.307 to 10.893) | <0.0001 | 0.74 |
